# Genome‐Resolved Approach of Guerrero Negro Hypersaline Microbial Mats Reveals the Metabolic Potential of Key Players in a Stratified Community

**DOI:** 10.1111/1462-2920.70199

**Published:** 2025-11-05

**Authors:** Miguel A. Martínez‐Mercado, Hever Latisnere‐Barragán, Patricia J. Ramírez‐Arenas, Ricardo Vázquez‐Juárez, José Q. García‐Maldonado, Alejandro López‐Cortés

**Affiliations:** ^1^ Programa de Planeación Ambiental y Conservación, Lab. Geomicrobiología y Biotecnología Centro de Investigaciones Biológicas del Noroeste S.C. (CIBNOR) La Paz Baja California Sur Mexico; ^2^ Centro de Investigación y de Estudios Avanzados del Instituto Politécnico Nacional (Cinvestav). Unidad Mérida Mérida Yucatán Mexico

**Keywords:** archaea, carbon cycle, hypersaline microbial mat, MAGs, methanogenesis, nitrogen cycle, sulfate reducing bacteria, sulfur cycle

## Abstract

Hypersaline microbial mats at Guerrero Negro harbor a stratified, highly diverse community with diel metabolic changes. While oxygenic photosynthesis and sulfate reduction are the dominant bacterial metabolic processes, methylotrophic methanogenesis is the main archaeal pathway. Although these metabolic processes have been biochemically characterized, the identity and encoded metabolism of the microorganisms have been inferred only from gene‐marker data. Here, a genome‐resolved approach in both environmental, as well as experimental dark condition samples (control, H_2_/CO_2_, TMA, and H_2_/CO_2_‐TMA) was used to stimulate less‐known anaerobic strategies, determine the metabolic potential of the main microbial players, and analyze the community. Representative metagenome‐assembled genomes (170 MAGs) were obtained, encompassing 25 bacterial and 4 archaeal phyla. The metabolic analyses of three basic elements (carbon, sulfur, nitrogen) encoded in the MAGs suggested that in environmental samples, phototrophic taxa were the main source of the organic matter that fueled most of the community. Different sulfur species acting as electron acceptors led to the metabolism of partially degraded organic matter in the lower layers of the mat. These results link and clarify the biochemical processes and microbial players, adding a novel genomic component for the ecological understanding of the microbial mats of Guerrero Negro.

## Graphical abstract

The first metagenome‐assembled genomes (MAGs) from Guerrero Negro hypersaline microbial mats were recovered from natural and incubated samples. These MAGs include dominant and rare taxa, whose metabolic potential provides clues to metabolic versatility in the cycling of three elements (carbon, sulfur, and nitrogen) at the genome and community levels. The MAGs' metabolic potential is consistent with their natural vertical distribution.

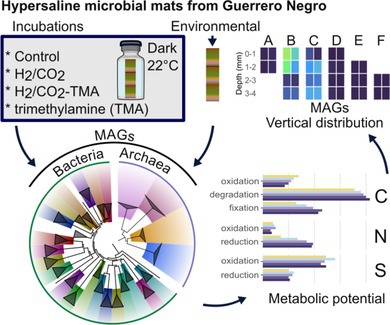

## 1 Introduction

Microbial mats are stratified organo‐sedimentary structures of microorganisms, mainly composed of bacterial, archaeal, and microeukaryotic populations with high metabolic diversity (Seckbach and Oren 2010). These communities occur in a variety of marine and freshwater environments (Bolhuis et al. 2014). However, the prevailing restrictive conditions of coastal intertidal zones promote the presence and persistence of microbial mats (Paerl and Yannarell 2010). The microbial mats developed within the seawater evaporation ponds of the Exportadora de Sal S. A., saltern in Guerrero Negro, Baja California Sur, Mexico, have been excellent model systems for microbial ecology research for more than 30 years (D'Amelio et al. 1987).

Characterisation of physicochemical processes in Guerrero Negro microbial mats has established a general biogeochemical pathway of organic and inorganic matter transformation (Canfield and Des Marais 1993; Des Marais 2003). During the day, photosynthesis by eukaryotic phototrophs (Feazel et al. 2008) and cyanobacteria (Ley et al. 2006) produces organic matter and oxygenation of surface layers. Oxygen concentration decreases with depth while hydrogen sulfide increases in concentration (Ley et al. 2006). This shift was thought to limit sulfate reduction and other anaerobic processes to the anoxic zone. However, sulfate reduction has been reported as an active process also observed in oxic layers of the mat during daytime (Canfield and Des Marais 1991). Hence, sulfate reduction can be considered one of the principal pathways involved in the organic carbon mineralization in the mat (Risatti et al. 1994). During the night, when photosynthesis and oxygenic conditions halt, together with the consumption of available oxygen, allow for the expansion of anoxic conditions from lower layers towards the surface of the mat, promoting the fermentation of the photosynthates (produced during the daytime), accompanied by hydrogen sulfide production (Des Marais 2003; Ley et al. 2006).

It has been determined that the methanogenic archaeal fraction in microbial mats is low, as the process that characterizes them is restricted to anaerobic conditions. The main methanogenic pathway is based on methylamines and methanol (García‐Maldonado et al. 2012; Kelley et al. 2015), leading to members of Methanosarcinales as the main group inhabiting this ecosystem. Other studies also described a wider diversity of archaeal lineages (Smith et al. 2008; Robertson et al. 2009), including all four known methanogenic pathways (García‐Maldonado et al. 2023).

Archaeal and bacterial communities in Guerrero Negro have been characterised through the use of marker genes (Ley et al. 2006; Harris et al. 2013; García‐Maldonado et al. 2015, 2018; Cadena et al. 2018; Ramírez‐Arenas et al. 2024), and more recently, a gene‐centric study of the metabolic potential in natural conditions explored nitrogen cycling (Maza‐Márquez et al. 2022), as well as the metabolic strategies developed as adaptations to the harsh hypersaline conditions (Maza‐Márquez et al. 2024). However, there are several gaps between our current understanding of active biogeochemical processes, the metabolic mechanisms, and the taxa that mediate them.

These studies determined that Guerrero Negro microbial mats are highly diverse and complex communities (Ley et al. 2006), containing representatives of more than 40 phyla (Harris et al. 2013). Cyanobacteria of the genera *Microcoleus, Leptolyngbya, Cyanobacterium, Synechococcus*, and *Spirulina* have been known to dominate in different evaporation ponds (Canfield and Des Marais 1993; Ley et al. 2006). Yet, Chloroflexota was determined to form the majority of biomass in the oxic zone (Ley et al. 2006). From the Desulfobacterota phylum, the genus *Desulfococcus* was determined to be the most abundant group in the photo‐oxic zone, while the genera *Desulfobacter* and *Desulfobacterium* were restricted to the suboxic zone (Risatti et al. 1994). Beyond these main processes, the microbiota of Guerrero Negro microbial mats is mostly unknown or described only as proposed *Candidatu*s lineages (Ley et al. 2006).

Here, a whole metagenome shotgun strategy followed by genome‐resolved analysis was used on Guerrero Negro hypersaline laminated microbial mats incubated with competitive or non‐competitive substrates to promote microorganisms with different metabolic potentials and with special focus on stimulating anaerobic metabolisms. The untreated environmental samples were also sequenced to determine the natural community structure. Our main aim was to recover bacterial and archaeal metagenome‐assembled genomes (MAGs) to reveal the metabolic strategies that would help explain their functional roles and rare‐taxa status in the natural community setting.

## 2 Experimental Procedures

### 2.1 Sampling, Incubation Experiments

In April of 2022, samples of 20 cm × 30 cm of laminated, soft‐smooth microbial mats were collected and maintained in site brine from two evaporation ponds named A4N5 (27°41′13.2“ N, 113°55′1.2” W) and A5 (27°41′24“ N, 113°55′1.2” W) at Exportadora de Sal, S.A. in Guerrero Negro, Baja California Sur, Mexico. Physicochemical environmental parameters were determined in situ, and the results were as follows for sites A4N5 and A5: 80‰ and 85‰ salinity, 20°C and 21.5°C, and pH 8.1 and 8.2. Then, seven cores (0.5 cm deep, 1 cm diameter) were subsampled. One batch of three cores per site was preserved in RNAlater (Thermo Fischer, USA), and stored at 4°C for transport and at −20°C until further processing. These untreated samples are hereafter called “environmental”. Seven additional cores (per treatment) were immersed in sterile artificial brine (80‰ salinity, 73 mM sulfate, pH 8.1), kept under dark conditions, and stored at room temperature for transport.

Microcosm experiments were used to evaluate the mat microbial community's response to substrate addition as described previously (Ramírez‐Arenas et al. 2024). Four treatments (each in triplicate) comprised a control incubation (no substrate added; N_2_ atmosphere), and incubations with the following conditions: H_2_/CO_2_ atmosphere (80%–20%) without substrate (named H_2_/CO_2_); H_2_/CO_2_ atmosphere (80%–20%) with trimethylamine (15 mM) as substrate (named H_2_/CO_2_‐TMA); and N_2_ atmosphere with trimethylamine (15 mM) as substrate (named TMA). Incubations proceeded in sterilised serum vials (50 mL) containing 20 mL of the treatment solution (artificial brine with or without substrate) and the seven microbial mat cores preserving their structural integrity. An anaerobic atmosphere was induced by capping the vials with butyl rubber stoppers and aluminium crimps. Then, the air was displaced with abundant N_2_ gas, followed by injection of the treatment atmosphere (N_2_ or H_2_/CO_2_) for two minutes. Vials were hermetically closed and incubated in the dark at 22°C for 85 days.

### 2.2 DNA Extraction and Whole Metagenome Sequencing

Total metagenomic DNA was extracted from approximately 2 g of each sample (environmental or incubated) with the DNeasy Power Biofilm kit (QIAGEN, Germany) with minor modifications in sample homogenization as described previously (Ramírez‐Arenas et al. 2024). A blank extraction (without a biological sample) was processed in parallel to assess possible cross‐contamination. The integrity of DNA was evaluated by visualization in 1% agarose electrophoresis and Nanodrop spectrophotometry (Thermo Scientific, USA). Three replicates per treatment were pooled, and samples were processed for library preparation and high‐throughput sequencing (2 × 150 bp) with a HiSeq machine (Illumina, USA) at Azenta Life Sciences (New Jersey, USA). Metagenomic raw sequencing data was deposited in NCBI's Sequencing Read Archive (https://www.ncbi.nlm.nih.gov/sra) under Bioproject PRJNA1086882.

### 2.3 Processing of Sequencing Data

Data for ten metagenomic libraries (from environmental and incubated samples) was received as raw fastq files. Initial processing included quality filtering and read trimming with Trimmomatic v.0.39 (Bolger et al. 2014) and removal of potential contaminants (human, PhiX, Lambda, UniVec database) with Fastq‐Screen v.0.15.3 (Wingett and Andrews 2018), retaining only paired reads. Comparison of k‐mer content among samples was done through sourmash 4.8.3 (Irber et al. 2022). First, low‐abundance k‐mers were removed with khmer v.3.0 (Crusoe et al. 2015). Then, signatures were sketched for k = 31 with a scaling of 10,000. After signature comparison, the result was analyzed through multidimensional scaling (MDS) and visualized in R 4.4 (R Development Core Team 2021) with the package sourmashconsumr (https://doi.org/10.57844/arcadia‐1896‐ke33).

Cleaned samples were assembled with Spades v.3.15.5 (Nurk et al. 2017) in ‘meta’ mode with a minimum contig length of 1000 nt. The resulting contigs were binned with SemiBin2 v.2.0.1 which employs a semi‐supervised siamese neural network (Pan et al. 2023). The multi‐binning advance workflow was used as it considers the difference in abundance among samples to generate bins for each sample analysed.

First, contigs from all ten samples were concatenated, and then the reads were mapped with bowtie2 v2.3.5 (Langmead and Salzberg 2012) retaining only mapped reads. Kmer composition and abundance data were obtained with the command ‘SemiBin2 generate_sequence_features_multi’ followed by training (SemiBin2 train_self) and application (SemiBin2 bin_short) of the auto‐supervised learning model to construct bins. Afterward, the quality of the bins was assessed with CheckM v.1.2.2 (Parks et al. 2015). All bins obtained were submitted together to dereplication with dRep v.3.5.0 (Olm et al. 2017). Species‐level Representative Genomes (as termed by dRep) were obtained with parameters: filtering of completeness 50, and contamination 25, MASH sketch size of 1000, fastANI algorithm (Jain et al. 2018), ANI threshold of 0.9 for primary clusters and 0.95 for secondary clusters—only bins of medium to high quality (Bowers et al. 2017) were used for further analysis.

Bins forming the species‐representative set are hereafter called MAGs. The MAGs were taxonomically classified with the Genome Taxonomic Database Toolkit gtdbtk v.2.1.1 (Chaumeil et al. 2022) and the GTDB database R207 (Parks et al. 2022). That taxonomic framework is used throughout the text and level prefixes (e.g., p_ for phylum) are indicated for clarity purposes as needed. The maximum‐likelihood trees inferred *de novo* with a set of 120 and 53 marker genes for bacteria and archaea respectively, were visualised in iTOL v.6 (Letunic and Bork 2024). The quantification of MAG abundance (trimmed mean of M‐values) in samples was calculated with CoverM v0.7.0 (Aroney et al. 2024) with reads recruited to MAGs by the “bwa‐mem” algorithm v0.7.18 (Li 2013). The MAGs with relative abundance > 1% in any given sample were defined as the dominant part of the community or top‐MAGs. This subset either represents naturally abundant taxa (environmental samples) or enriched taxa (treatment samples). A Procrustes analysis and a Procrustes randomization test (vegan's protest function, permutations = 999) were used to compare the configurations between the unassembled data (k‐mer level, described above) and the MAGs (log‐transformed abundances, Euclidean distances between samples). The first two dimensions of the rotation were visualised in an ordination diagram.

Functional annotation of MAGs was obtained with DRAM v1.5 (Shaffer et al. 2020). Additionally, METABOLIC v4 (Zhou et al. 2022) was used to infer functional contributions and metabolic weight scores (MW‐scores) for the top‐MAGs compared against each sample. MW‐scores are a community‐level metric combining the functional capacity with the abundance of the microorganisms encoding it in co‐sharing functional networks (Zhou et al. 2022).

The functional annotations were revised manually to confirm and categorize the potential of encoded metabolisms of interest. First, a set of genes involved in the different carbon fixation pathways was used to identify autotrophic categories (Garritano et al. 2022): Calvin Benson‐Bashan (CBB), reverse tricarboxylic acid cycle (rTCA), 3‐hydroxypropionate bi‐cycle (3HP), 3‐hydroxypropionate/4‐hydroxybutyrate (HP/HB), dicarboxylate/4‐hydroxybutyrate (DC/HB) cycle, and the Wood–Ljungdahl pathway (WLP). All the other genomes were considered heterotrophic. Other functional annotations focused on the main biogeochemical processes of carbon, nitrogen, and sulfur, as well as carbohydrate‐active enzymes (cazymes), peptidases, and enzymes and complexes involved in electron transfer, were also evaluated.

An overview of the community was generated by identifying 16S rRNA gene sequences in contigs with barrnap 0.9 (Seemann 2013), quantified in the metagenomes with salmon (Patro et al. 2017). Then OTUs (97%) were formed with VSEARCH (Torognes et al. 2016), and taxonomic assignment used the GTDB R214 database formatted for dada2 (https://doi.org/10.5281/zenodo.10403693).

### 2.4 Vertical Distribution

A public metagenome dataset from a nearby site (Maza‐Márquez et al. 2022) was used to evaluate the presence and vertical distribution at the millimetre scale of the MAGs obtained in this study. The dataset comes from a Guerrero Negro microbial mat sampled in 2019 at concentration pond Area 4. That sample was sliced at one‐millimetre intervals corresponding to depths 0‐1 mm (L1), 1–2 mm (L2), 2–3 mm (L3), 3–4 mm (L4). Raw metagenomic reads from the four samples were downloaded and subjected to quality control with Trimmomatic, followed by low‐abundance kmer cleaning (khmer). Finally, signatures were calculated using “sourmash”. The MAGs‐kmer signatures were then used to quantify (sourmash function gather) the average genome coverage (proxy for abundance) in each of the four depths. Additionally, MW‐scores of the top‐MAGs present in the millimetre scale dataset were calculated as described above.

## 3 Results

### 3.1 Sequencing and Data Summary

The microbial community of hypersaline microbial mats from Exportadora de Sal, S.A., at Guerrero Negro sites A4N5 and A5 was analysed from environmental samples and microcosm incubations under dark conditions and with substrate additions. Metagenomic data analysis focused on recovering the genomes of the naturally dominant members and those stimulated by the treatments, and investigating their individual metabolic potential and inferring their contribution to the community. In the set of analysed samples, the mean number of reads that passed quality control for samples from A4N5 and A5 was 40.75 and 39.68 million, respectively. The assemblies consisted of an average of 207,001 and 192,975contigs (length > 1000 bp), for samples from sites A4N5 or A5. Table S1 summarises processing of sequences by sample.

**FIGURE 1:**
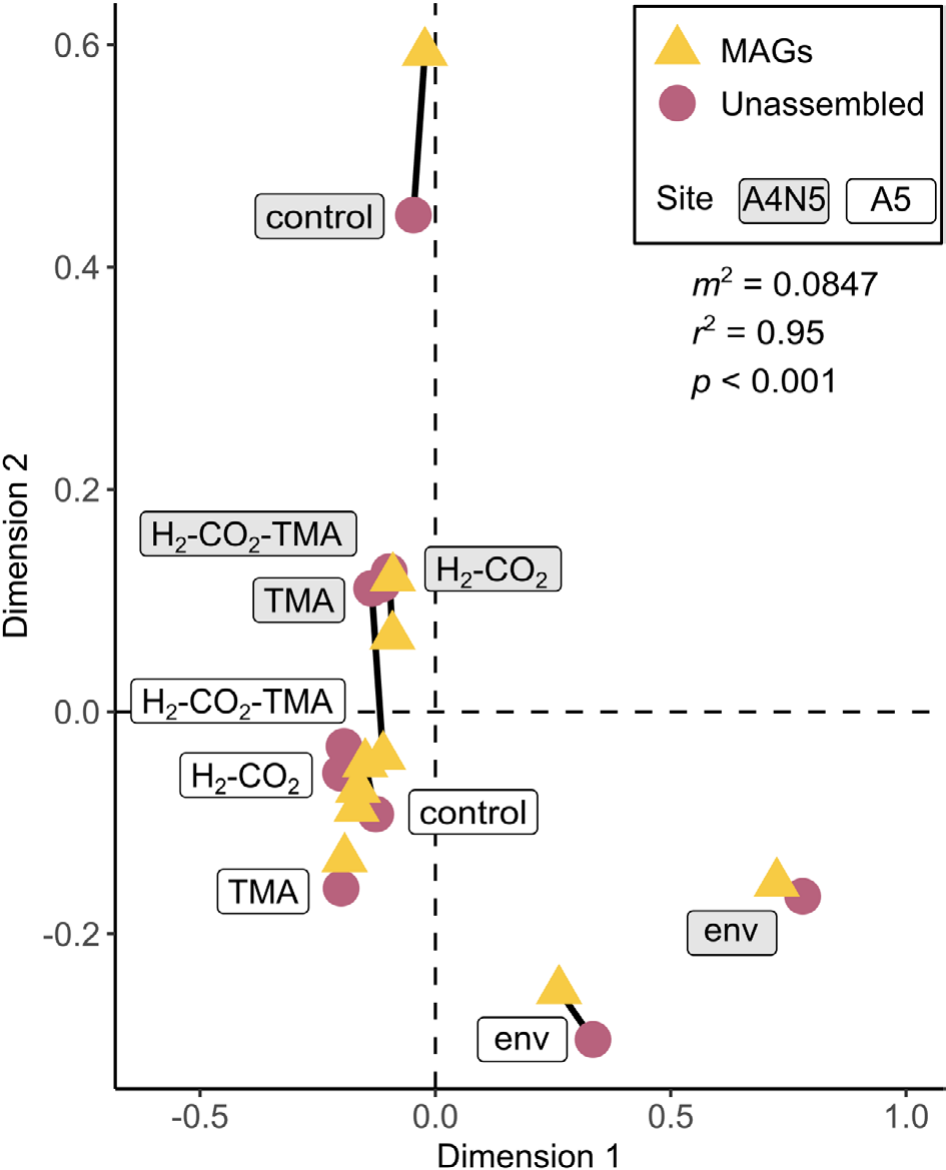
Procrustes analysis between unassembled data and MAGs. Circles show the community configuration based on k‐mer data, and are connected through lines to triangles representing sample positions based on MAGs after the Procrustes rotation. Sample types (environmental or treatments) are shown as labels. Statistics from the analysis: Procrustes rotation (*m* ^*2*^), correlation coefficient (*r* ^*2*^), and *p*‐value are shown inside the plot.

The effect of the substrate addition experiments on the community was evaluated at the k‐mer level. Coupled with ordination analysis, the results show the separation of the environmental samples from those subjected to incubations (Figure 1, first component in Figure S1) indicating the change of the community structure. A second separation of samples was owing to their site of origin (Figure 1, and Figure S1). Moreover, samples corresponding to the control and H_2_/CO_2_ treatments were the closest in similarity, compared to the other treated samples of site A4N5, while H_2_/CO_2_ and TMA treatments were the closest for site A5. These results indicate an effective change in community structure induced by the incubations, although without a clear effect associated with a specific substrate.

In the subsequent data processing, genome binning yielded 1650 bins that were dereplicated at the species level into 170 medium to high‐quality MAGs. Of these, 163 were assigned to the domain Bacteria, and 7 to Archaea. Approximately 23% of contigs (of length > 1000 bp) per sample were used in bins; they account for an average of 58.11% (min 54.62%, max 61.6%) of the reads mapping to contigs (Table S1). The community structure represented by the MAGs has a significant superimposition (Procrustes sum of squares = 0.0847, *p* < 0.001) of high correlation (r^2^ = 0.95) with the data at the k‐mer level (Figure 1). This result indicates that the MAGs provide a robust representation of the community present in the unassembled data, and the community modifications associated with the incubations.

### 3.2 MAGs From a Highly Diverse Community

Extraction of 16S rRNA gene sequences from the metagenome showed a highly diverse community composed of 42 bacterial and 7 archaeal phyla (Table S2). Of those, eighteen phyla had relative abundance > 1% in at least one sample (Figure S2). The incubated samples contained phyla not detected in the environmental sample as well as phyla with increased abundance (Table S2) supporting the community modifications observed previously.

**FIGURE 2:**
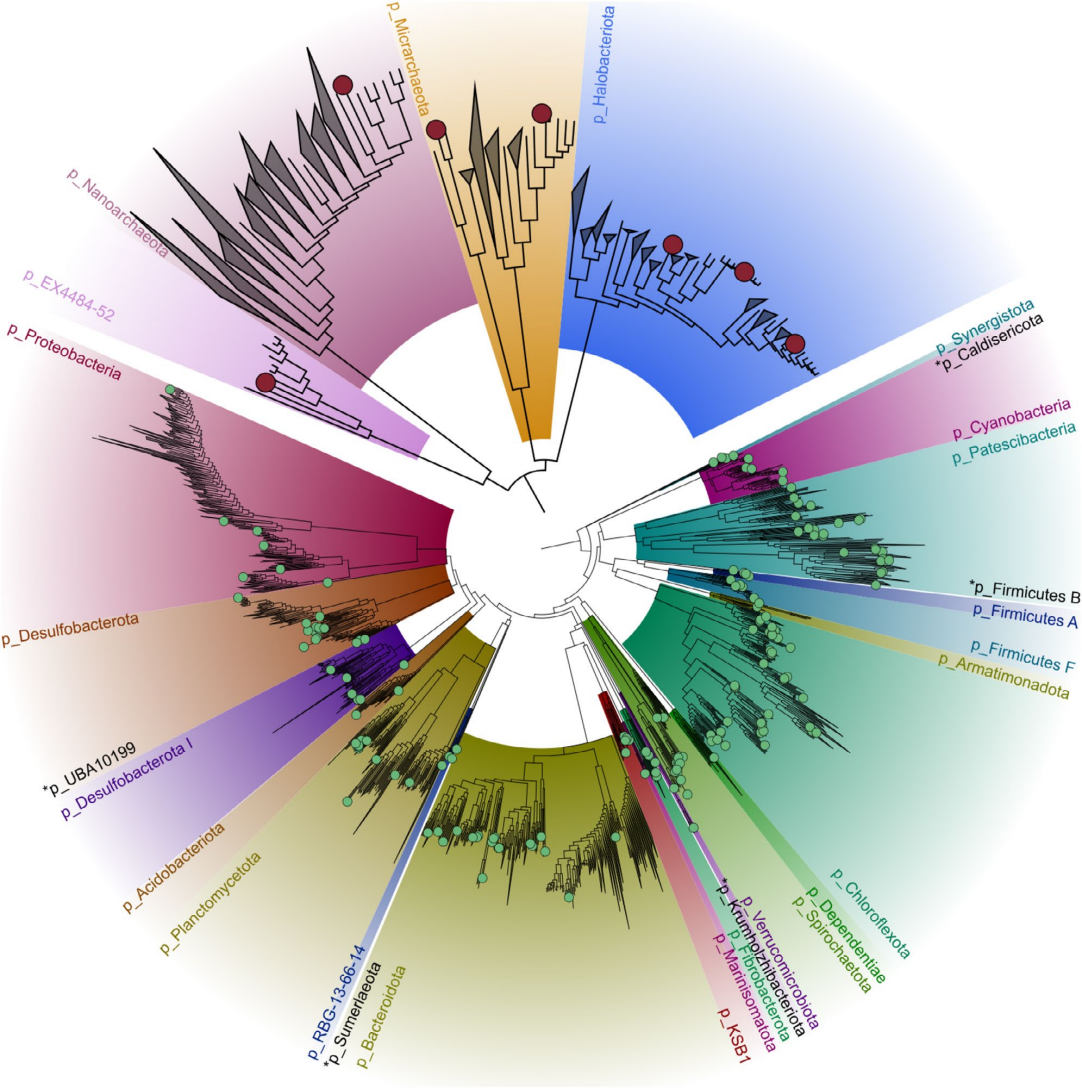
Phylogenomic analysis of the Guerrero Negro metagenome assembled genomes (MAGs). Maximum‐Likelihood *de novo* trees based on concatenated 120 bacterial or 53 archaeal single‐copy genes. The location of bacterial (163) and archaeal (7) MAGs are shown in green and red circles, respectively. Clades are coloured at the phylum (p_) level following GTDB version R207. Phyla with MAG representatives but with insufficient space to display a coloured area are indicated with a star.

The genome‐resolved strategy recovered bacterial MAGs belonging to 25 phyla, while the archaeal MAGs belonged to 4 phyla (Figure 2). The phylum Chloroflexota had the highest number of recovered MAGs, with 40 representatives, followed by Patescibacteria (24 MAGs), Bacteroidota (20 MAGs), and Desulfobacterota (15 MAGs) (Figure 2, Table S3). For Archaea, seven representative MAGs were recovered from phyla: Halobacteriota (3 MAGs), Micrarchaeota (2 MAGs), Nanoarchaeota (1 MAG) and EX4484‐52 (1 MAG) (Figure 2, Table S3). Of note, the 25 phyla represented by MAGs include 17 out of the 18 phyla with relative abundance > 1% identified through 16S, indicating that dominant and rare members of the community were recovered.

At the family level, 104 taxa were identified by analysing 120 bacterial or 53 archaeal single‐copy genes. The MAGs dataset represented great novelty at the family level, with only 35 out of 104 taxa (33%) having a name (i.e., already described), 50 (48%) named with a code (i.e., reported genome, but undescribed taxon), and 19 (18%) lacking assignment (Table S3, Figure S3). At a finer taxonomic scale (genus or species levels), 109 out of the 170 (64%) MAGs lack taxonomic assignment (Table S3). Among the few MAGs that achieved assignment to (a described) species level was E22bin.1611 as *Thiohalocapsa halophila* (f_Chromatiaceae) and E22bin.1561 as *Salinivirga cyanobacteriivorans* (f_Salinivirgaceae).

Estimation of diversity based on MAG's (*n* = 170) relative abundances resulted in a Shannon index ranging from 4.13 to 4.51 (Table S1). The most diverse samples by site were the control (Shannon index = 4.51) and H_2_/CO_2_ (Shannon index = 4.46) incubations from A4N5 and A5, respectively. On the other hand, Pielou's evenness values indicated that the environmental (A4N5 = 0.8279, A5 = 0.8357) and the TMA treatment (A4N5 = 0.8328, A5 = 0.8142) samples showed a slight trend towards lower evenness (Table S1), suggesting that a few taxa become dominant under these conditions.

A total of 76 MAGs surpassed 1% of relative abundance in a given sample and were classified as naturally dominant or as the favourably stimulated members in the treatment samples. These top MAGs were associated with 23 phyla (out of 29 detected, or 79%). Of note, the top MAGs included members of the four archaeal phyla identified, indicating that none of the archaeal groups were excluded by the abundance filter.

### 3.3 Microcosm Incubations Modify the Community Composition

**FIGURE 3:**
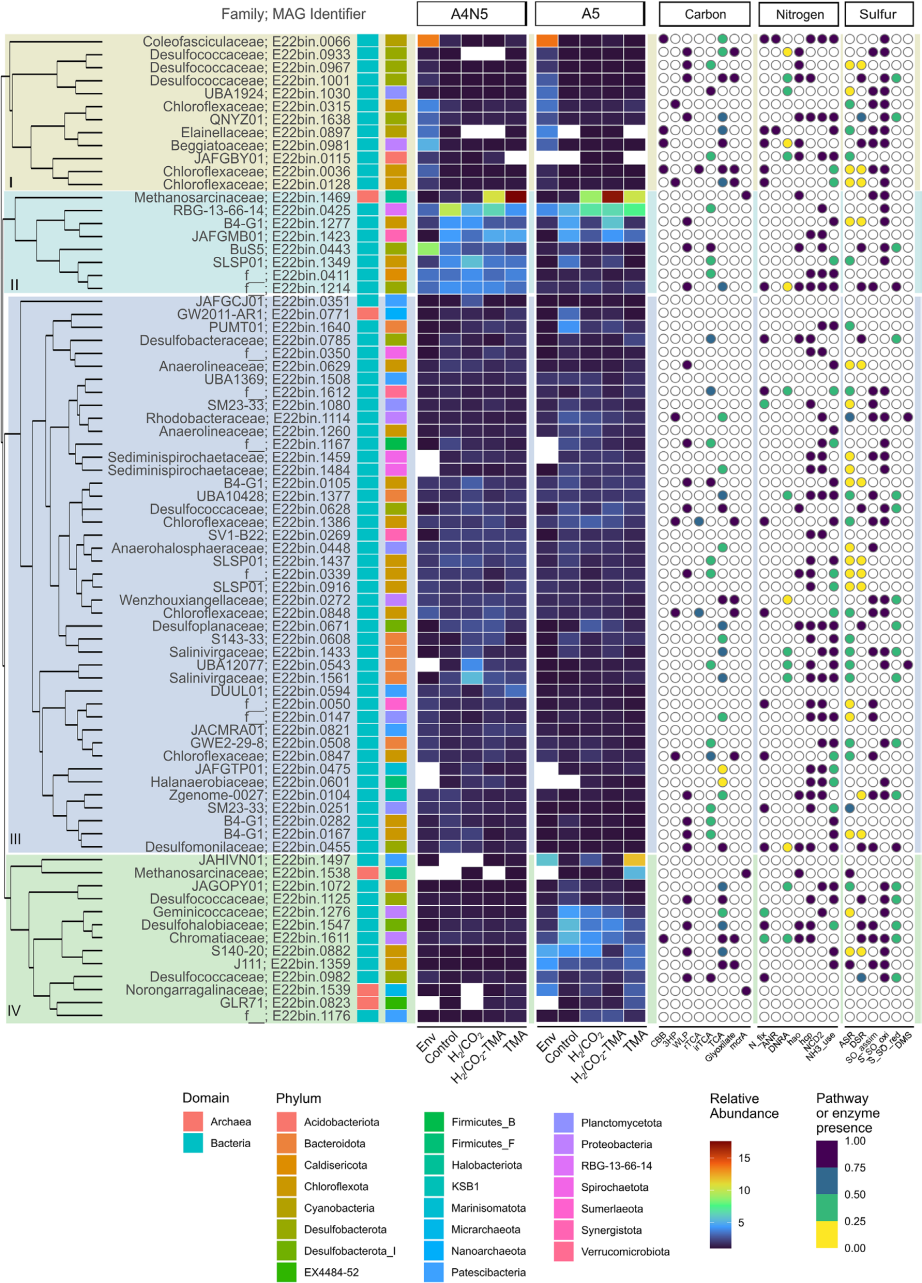
Top abundant MAGs. The central heatmap shows the relative abundances of the top abundant MAGs (> 1% per sample) at each site. The cladogram at the left shows the result of a hierarchical cluster analysis on the MAG abundances, with four clusters highlighted (I to IV). Treatments: Env (environmental), Control, H_2_/CO_2_, H_2_/CO_2_‐TMA, TMA. TMA = trimethylamine. The panel on the right shows the presence of genes or the extent to which completion of the three main element pathways (carbon, nitrogen, sulfur), were achieved. White circles in the abundance heatmap or the genes/pathways indicate absence.

A cluster analysis of the top‐MAGs' relative abundances formed 4 clusters (named I to IV). These clusters show the effect of microcosm incubations over groups of taxa common to sites or specific to each site (Figure 3). Cluster I was composed of twelve MAGs with higher relative abundance in environmental samples and mainly with potential for light‐driven metabolisms or dependent on the products of this metabolism. Shared MAGs between both sites were the cyanobacterial families Coleofasciculaceae (E22bin.0066) and Elainellaceae (E22bin.0897); the gammaproteobacteria Beggiatoaceae (E22bin.0981), and one member of the green non‐sulfur bacterial family Chloroflexaceae (E22bin.0036), all suggested as capable of carbon fixation by CBB (*rbcL*, *rbcS*, *prk*) and nitrogen fixation (*nifD*, *nifH*, *nifK*). The inferred sulfur‐oxidising MAG (E22bin.0981) associated with Beggiatoaceae indeed contained a wide machinery for sulfur‐compound oxidation: thiosulfate to sulfate (*sox*), thiosulfate to sulfite (*sseA*), sulfide to sulfur (*fccAB*), sulfide to sulfite (*pdo*, *sqr*), sulfite to sulfate (*soeABC*), thiosulfate to tetrathionate (*tsdA*) (Table S4). Additionally, this MAG also showed genes related to dissimilatory sulfate reduction (*sat*, *aprAB*, *dsrAB*). A Chloroflexaceae (E22bin.0315) and one undescribed Desulfobacterota (family QNYZ01, E22bin.1638) with capacity for different carbon fixation strategies (3HP and WLP, respectively), were also top‐MAGs common to both sites (Figure 3).

Cluster II contained MAGs with the highest abundances in the substrate incubations, preferentially in A4N5 (Figure 3). Taxa with top‐MAGs across the A4N5 site belonged to families: B4‐G1 (p_Chloroflexota, E22bin.1277), BuS5 (p_Desulfobacterota, E22bin.0443), both taxa capable of using the WLP pathway for carbon fixation; SLSP01 (p_Chloroflexota, E22bin.1349), and RBG‐13‐66‐14 (E22bin.0425) encoding sulfur and thiosulfate oxidation; Caldisericota (E22bin.0411) and Synergistota (E22bin.1423) MAGs lacked genes for carbon fixation or sulfur cycling but encoded genes for hydroxylamine processing (Figure 3, Table S4), and a MAG from an unknown family within phylum Desulfobacterota (o_Desulfomonilales, E22bin.1214) with a versatile metabolic potential including WLP, nitrogen fixation and sulfate, sulfur and thiosulfate reduction. Of note, cluster II included an archaeal MAG associated with methanogenic archaea of family Methanosarcinaceae (E22bin.1469) and with enriched presence in incubations containing TMA for both sites (Figure 3).

Cluster III included the top‐MAGs with a rather homogeneous low abundance (just over the 1% cutoff) or enriched in specific samples or sites (Figure 3). Examples of the former pattern are inferred heterotrophic MAGs: Anaerohalosphaeraceae (E22bin.0448), SLSP01 (E22bin.1437), and a potential sulfate reducer Desulfoplanaceae (E22bin.0671). A contrasting pattern was observed in taxa Salinivirgaceae with MAG E22bin.1433 having a low abundance, while E22bin.1561 was enriched only in the A4N5‐H_2_/CO_2_ sample, suggesting other metabolic differences than the presence of genes for sulfur and thiosulfate oxidation. MAGs encoding 3HP cycle (Chloroflexaceae: E22bin.1386, E22bin.0847, and E22bin.0848; Rhodobacteraceae: E22bin.1114) and WLP (Anaerolineaceae: E22bin.0629; B4‐G1, E22bin.0105, E22bin.0167, E22bin.0282; Desulfococcaceae: E22bin.0628; and Desulfomonilaceae: E22bin.0455) pathways were also observed in this cluster.

The cluster IV contained taxa with enrichment only in A5 samples (incubated or not) (Figure 3). Taxa belonged to the heterotrophic family Geminicoccaceae (E22bin.1276), and encoded carbon‐fixing Chromatiaceae (CBB; E22bin.1611), Desulfohalobiaceae (WLP; E22bin.1547), S140‐20 (WLP; E22bin.0882), Desulfococcaceae (WLP; E22bin.0982, E22bin.1125). Cluster IV also included three out of five archaeal top‐MAGs, associated with families: GLR71 (p_EX4484‐52, E22bin.0823), Methanosarcinaceae (E22bin.1538), and Norongarragalinaceae (p_Micrarchaeota, E22bin.1539). Of the two archaeal MAGs with an *mcrA* gene in cluster IV, only Methanosarcinaceae is a methanogenic lineage. This MAG was enriched only in the samples with the addition of TMA (Figure 3).

The clusters formed with the MAGs relative abundance data do not show a clear association with the metabolic potential of the three main elements analysed (C, N, S). As observed in Figure 3, only cluster I was apparently associated with CBB carbon fixation, but cluster IV also contained a MAG with this capability. Furthermore, all MAGs of the Patescibacteria phylum lacked the selected genes and pathways (Figure 3, Figure S4). This lineage was present in clusters III (DUUL01, E22bin.0594; JACMRA01, E22bin.0821; JAFGCJ01, E22bin.0351; UBA1369, E22bin. 1508) and IV (GLR71, E22bin.0823; JAHIVN01, E22bin.1497; and f_, E22bin.1176). These results suggest that other genes, pathways, or a combination of these may be of more importance in shaping the community structure under the tested conditions.

### 3.4 Shifts in Metabolic Potential

**FIGURE 4:**
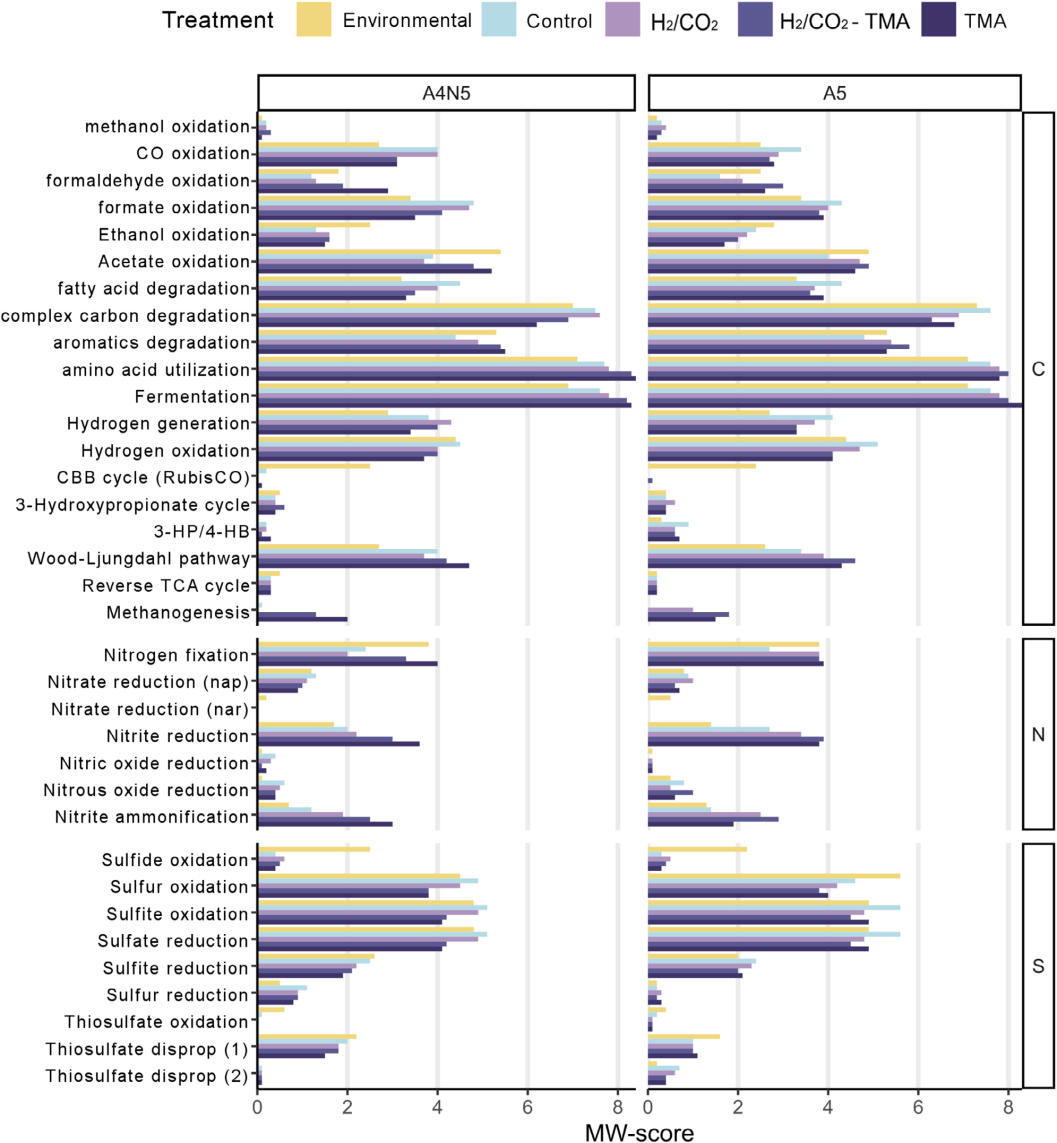
Metabolic profile of microbial communities. Metabolic weight scores (MW‐scores) were obtained with METABOLIC software by analysing the set of top‐MAGs of environmental or incubated samples. The MW‐scores are shown for the three main biogeochemical elements (carbon, nitrogen, sulfur). TMA = trimethylamine.

The top‐MAGs were used to assess the putative metabolic capacity of the communities derived from the MW‐scores (Figure 4). The environmental samples were characterised by high MW‐score values (> 2) in carbon fixation (CBB and WLP), nitrogen fixation, and associated with sulfur cycling processes like sulfate and sulfite reduction, as well as sulfide, sulfur, and sulfite oxidation. In addition, other potential carbon fixation pathways were observed (3HP, rTCA), although with a minor contribution.

The control incubation likely halted the CBB carbon fixation, and reduced the nitrogen fixation and nitrate reduction; it could also have decreased several oxidation processes such as formaldehyde, ethanol, acetate, sulfide, sulfur, and thiosulfate (Figure 4). On the other hand, an increase was observed in the potential for carbon fixation by WLP, fatty acid degradation, complex carbon, and amino acid utilisation: fermentation, hydrogen generation and oxidation, formate oxidation, sulfate reduction, and sulfite oxidation. Dark and anaerobic conditions presumably stopped the generation of photosynthesis products, and the previously existing organic matter (mainly cyanobacterial biomass) was likely made available for consumption.

Similar to the control incubation, the other treatments showed that WLP possibly continued as the main carbon fixation pathway. However, compared to control, the addition of substrates probably reestablished and sometimes likely augmented the capability of metabolisms found in environmental conditions, especially those related to carbon and nitrogen cycling. The augmented capabilities would include fermentation, amino acid degradation, formaldehyde oxidation, nitrogen fixation, nitrous oxide reduction, nitrite reduction, and nitrite ammonification. Most of the sulfur processes were presumably negatively affected (except sulfur reduction), as well as the degradation of fatty acids, complex carbon, ethanol and hydrogen oxidation (Figure 4). As hypothesized, there was a notable increase in the potential for methanogenesis by the addition of TMA alone or in combination with H_2_/CO_2_. In the A5 sample, the addition of H_2_/CO_2_ may have also stimulated methanogenesis. It is worth noting that genes used for aerobic methanotrophy (*pmmo*, *smmo*) were not identified in any of the treatment samples when considering the top‐MAGs (or the full 170 MAG dataset, not shown).

Potential for degradation of complex organic matter driven by genes for carbohydrate‐active enzymes (cazymes) is highlighted here for families of glycosyl hydrolases (GHs), polysaccharide lyases (PLs) and carbohydrate esterases (CE) (Figure S5). The topMAGs encoding an above‐average (> 16) diversity of cazyme GH families also possessed relatively high diversity of PL and CE families. These taxa were associated with Bacteroidota (UBA10428, E22bin.1377; PUMT01, E22bin.1640), Chloroflexota (SLSP01, E22bin.1437, E22bin.1349, E22bin.0916; Anaerolineaceae, E22bin.1260, E22bin.0629), KSB1 (E22bin.0104), Marinisomatota (E22bin.0475), Planctomycetota (SM23‐33, E22bin.1080; Anaerohalosphaeraceae, E22bin.0448), Spirochaetota (Sediminispirochaetaceae, E22bin.1459), Sumerlaeota (E22bin.0050), and Verrucomicrobiota (E22bin.1612). Accordingly, the inferred cazyme profile of these taxa had diverse target substrates (Table S5). Recurrent among topMAGs were GHs targeting the backbone of starch (GH13, GH57, GH77), chitin (GH18, GH23), mucin (GH109), xylan and xyloglucan (GH2, GH5, GH31, GH39, GH74, GH133), pectin (GH4, GH28, GH106), trehalose (GH15, GH37, GH65), agar (GH16), cellobiose (GH94), mannobiose (GH130) among other compounds and linkages. Potential PL substrates included pectin (PL1, PL9, PL11, PL22), sulfo‐polysaccharides (PL12, PL29, PL33), alginate (PL6, PL14, PL15, PL31, PL34), chondroitin (PL35), and rhamnose (PL42). While inferred CEs were mainly directed to the deacetylation of polysaccharides (ce1, ce4, ce9, ce7, ce11, ce14) and peptidoglycans (ce4, ce9).

Peptidases are also helpful enzymes in the degradation of organic matter. Genes encoding eight peptidase types were found in the topMAGs (Figure S6, Table S6). MAGs associated with phyla KSB1 (E22bin.0104), Cyanobacteria (E22bin.0066) and Chloroflexota (E22bin.0167, E22bin.0916, E22bin.1359, E22bin.0882) contained around 300 peptidase genes.

Overall, the results suggested that in both environmental samples and in samples with substrate additions, there may exist a heavy reliance on the metabolism of complex carbon, amino acids, and fermentation processes (Figure 4).

### 3.5 Vertical Distribution of MAGs

**FIGURE 5:**
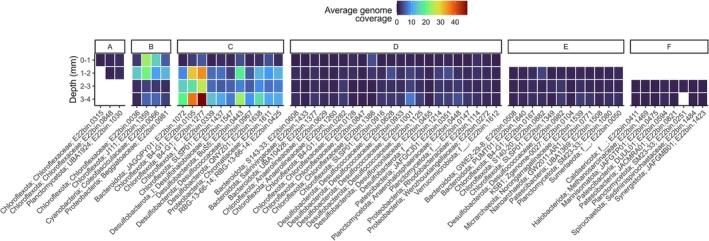
Vertical distribution of top‐MAGs. Kmer‐based quantification of top‐MAGs on millimetre‐scale mat samples (Maza‐Márquez et al. 2022). The heatmap shows the genome average coverage and white spaces indicate genome absence. MAGs are grouped by their vertical distribution profile (A to F). Bottom labels indicate phylum, family and MAG id.

The presence and abundance (genome average coverage) of the top‐MAGs were assessed in a public millimetre‐scale metagenomic dataset from intact microbial mats in site A4N5, revealing their natural vertical stratification within the surficial 4 mm. At the mm‐scale, the first two layers (0–2 mm) comprise the oxic zone, the third layer (2–3 mm) represents a transition zone, and the fourth layer (3–4 mm) is mostly anoxic. Based on k‐mer analysis, sixty (out of 76%, or 79%) top‐MAGs were detected (Figure 5), and their vertical distribution was grouped into six distinct profiles (named A‐F).

MAGs present only in the oxic zone (profile A) represented two potential autotrophs (3HP pathway) from Chloroflexaceae and one potential heterotroph from the phylum Planctomycetota. Almost half of the MAGs detected (*n* = 36) were present across the four layers (profiles B, C, and D), but differing in abundance. Three potential autotrophs also capable of nitrogen fixing, from families Chloroflexaceae, Coleofasciculaceae, and Beggiatoaceae, and a fourth MAG, possibly a heterotroph from family J111 (p_Chloroflexota), displayed higher abundance in the oxic zone (profile B). Eleven MAGs with increasing abundance below the surface layer (profile C) were associated with potential autotrophs from phyla Desulfobacterota and Chloroflexota encoding WLP, and *Thiohalocapsa halophila* (f_Chromatiaceae) encoding CBB. The most prevalent distribution profile (D) was observed in 22 MAGs, including representatives of 16 families with several members of the phyla Chloroflexota and Desulfobacterota. MAGs with presence in all but the first layer (profile E) were mostly associated with potential heterotrophs. Of note, archaeal MAGs from phyla Nanoarchaeota and Micrarchaeota displayed this vertical distribution profile. Lastly, MAGs restricted to the two bottom layers (profile E) represented the archaeal family Methanosarcinaceae, and the bacterial phyla Caldisericota, Marinisomatota, Patescibacteria, Planctomycetes, Spirochaetota, and Synergistota.

For a microorganism to be present across the four layers (Figure 5, profiles B‐D), it may require possessing a very versatile metabolism in order to endure the shifting conditions. Alternatively, a microorganism may change its vertical position towards better conditions. In fact, different motility mechanisms were identified in topMAGs (Table S4). Gliding motility genes (*gld*) were the most common, identified in 54 MAGs, often accompanied by different sets of chemosensory genes (*che*, *mcp*, and *wsp* genes). Flagellar motility was the second potential mechanism with an occurrence in 17 bacterial topMAGs (*fli* genes) and 2 archaeal topMAGs (*Fla* genes). A set of genes for twitching (*pil*) was present in Beggiatoaceae and Wenzhouxiangellaceae MAGs, and an incomplete set was identified in topMAGs of families J111 (Chloroflexota), Coleofasciculaceae, Elainellaceae (Cyanobacteria), BuS5, and Desulfococcaceae (Desulfobacterota). Phototaxis genes (*pix*) were exclusive to cyanobacteria. On the opposite side, several taxa, including Caldisericota, RBG‐13‐66‐14, and most Patescibacteria topMAGs, lacked any of the previously mentioned strategies for potential motility. These results suggest that most community members' distribution across layers is not fixed, and may be due to adaptations to a stratified and constantly changing environment.

### 3.6 Role of Top Players in the Stratified Community

**FIGURE 6:**
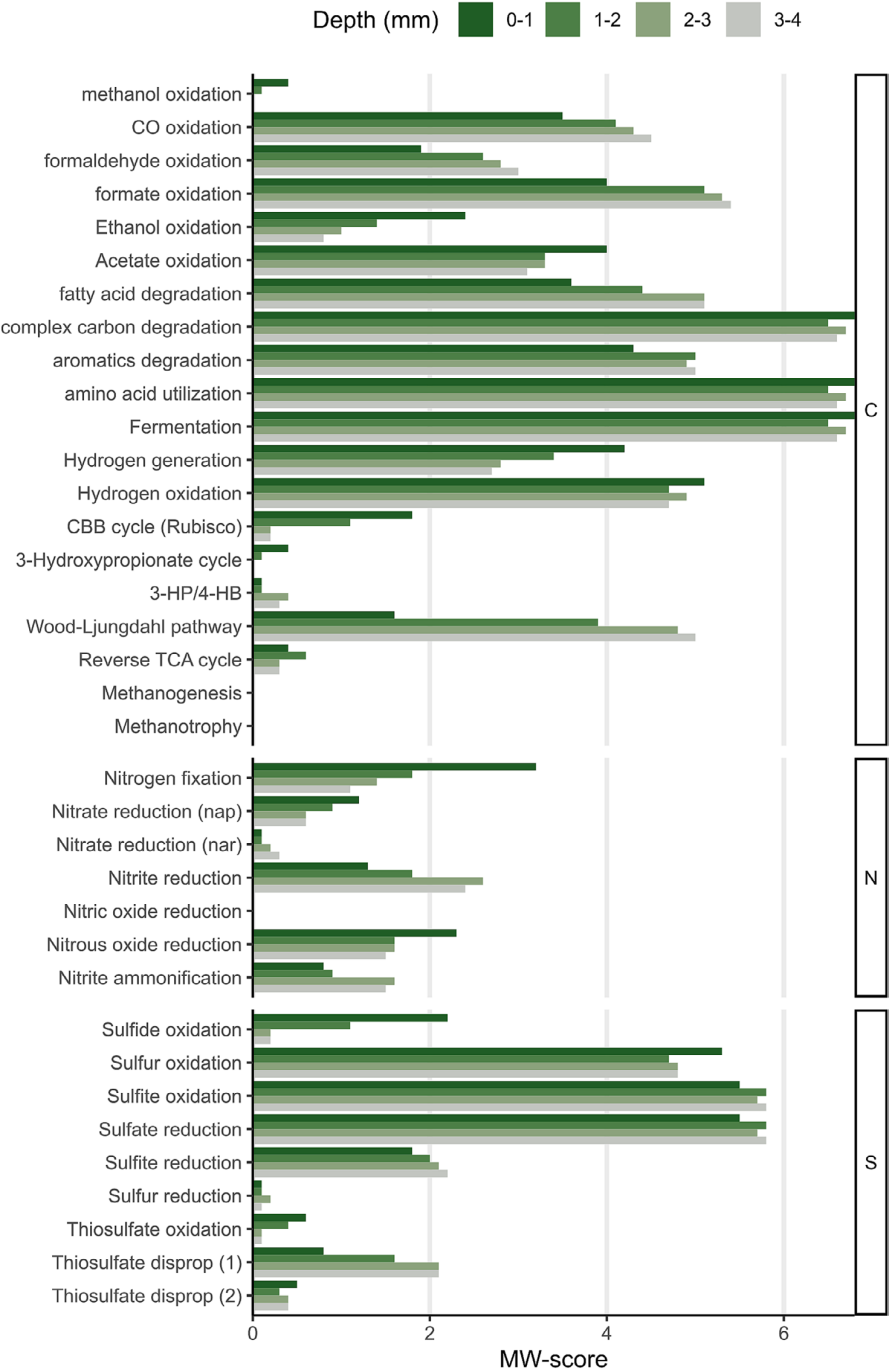
Metabolic profile of environmental samples. Metabolic profile of MAGs in an environmental four‐layered sample. Metabolic weight scores (MW‐scores) obtained with METABOLIC software by the analysis of the top‐MAGs set recovered in this study, and compared against the metagenomic data of the millimetre‐scale samples. The mw‐scores are shown for the three main biogeochemical elements: carbon (C), nitrogen (N), and sulfur (S). disprop = disproportionation.

The breakdown of MAG's metabolic capabilities across the vertical layers showed a similar profile of mw‐scores (Figure 6) to that of the A4N5 and A5 environmental samples (Figure 4). The profile of metabolic potential is indicative of a community likely relying on the degradation of complex carbon, amino acids, and fermentation (Figure 6). The other possibly fundamental metabolism was related to sulfate reduction, but not to sulfite reduction, suggesting different uses for sulfate reduction. Although the majority of functions were likely present across the layers, there were specific pathways with higher representation in specific sections of the mat.

Potential for carbon fixation through the CBB cycle was characteristic of the upper layers (0–2 mm). In minor proportion but distinctive of these layers was possibly the 3HP cycle. The oxidation of alcohols (methanol and ethanol) and hydrogen generation were probably also preferential in these layers. Other important potential metabolic pathways included sulfide and thiosulfate oxidation, nitrogen fixation, and nitrous oxide reduction (Figure 6). The last two probably occur during nighttime. Overall, the distinctive metabolic potential of the upper layers points towards oxygenic and anoxygenic photosynthesis.

The metabolism of the bottom layers stands out for the possible use of WLP for carbon fixation, oxidation of C1 compounds (CO, formate, formaldehyde), degradation of fatty acids, reduction and ammonification of nitrite (Figure 6). Thiosulfate disproportionation (through *phsA*, forming sulfide and sulfite) was likely a relevant sulfur transformation. This potential metabolic profile fits well with an anaerobic community in a sulfate‐rich environment, with growth likely based on organic carbon compounds as electron donors and sulfur compounds as electron acceptors.

## 4 Discussion

### 4.1 First Genome‐Based Analysis of Guerrero Negro Microbial Mats

Community composition in microbial mats varies according to the location where they develop (Prieto‐Barajas et al. 2018; Wong et al. 2016). In the highly diverse microbial mats of Guerrero Negro, more than 40 phyla have been identified (Ley et al. 2006; Harris et al. 2013; García‐Maldonado et al. 2023), making them difficult to approach by genome‐based strategies due to the sequencing effort required to cover beyond the dominant members (Kunin et al. 2008; Pascoal et al. 2021). Thus far, no genomes have been recovered from Guerrero Negro beyond cyanobacterial cultures (García‐Pichel et al. 1998) and other untracked Methanosarcinaceae enrichments (Jahnke et al. 2008). This study recovered MAGs from 29 phyla. The phyla represented in the MAGs are in agreement with the previous reports based on 16S amplicon sequencing (Ley et al. 2006; Harris et al. 2013) and gene‐centered metagenomics (García‐Maldonado et al. 2023; Maza‐Márquez et al. 2024) by covering the dominant phyla as well as several low‐abundant members. These MAGs were recovered from both environmental samples and microcosm assays incubated with competitive and non‐competitive substrates. Hence, the substrate addition experiments were also useful to yield MAGs other than the naturally dominant community members.

The novelty of the recovered genomes was determined by the taxonomy assigned with a set of single‐copy genes, which was reached mostly at the family level. In fact, the closest genomes were associated with similar or extreme environments, such as hypersaline soils, salt pans, freshwater, marine coastal and deep‐sea sediments, and hydrothermal vents (Murphy et al. 2021; Tran et al. 2021; Vavourakis et al. 2018; Zhou et al. 2022). The closest reference genomes usually belonged to uncultivated, undescribed lineages, known only by their genome sequence, highlighting how culture‐independent strategies become important for microbial diversity studies.

A broader diversity was also observed in previously recognised phyla. For instance, the importance of sulfate reducers in microbial mats is well known, and different lineages have been previously analysed either by gene markers or targeted by enzymes involved in the process (Risatti et al. 1994; Cadena et al. 2018). However, a finer taxonomic and diversity resolution was achieved with at least 12 families (two unknown) represented. For instance, within the family Desulfococcaceae, six potentially different species were detected. Although the six MAGs likely based their carbon fixation on the WLP pathway, differences in other pathways were observed, like differential TCA cycle components, the capability for nitrogen fixation, or sulfur reduction. Similarly, potential phototrophic lineages within the Chloroflexota were also diverse as ten families were detected. The Chloroflexaceae family was represented by nine different taxa (MAGs) with differences in genetic content, probably leading to functional differences in carbon and nitrogen fixation, and processing of sulfur compounds. Overall, the MAG diversity supported and replicated the known microbial mat community diversity and its structure (Spear et al. 2003; Ley et al. 2006; Harris et al. 2013). With the vertical distribution approach, a highly diverse community, dominated by Cyanobacteria and Chloroflexota in the oxic layer followed by Proteobacteria, Bacteroidota, and Desulfobacterota in the layers below, was recognised.

A cutoff of 1% relative abundance was selected to define the top members. This filter dismissed 94 MAGs. Given that these MAGs were naturally in low abundance and that none of the incubations tested increased their abundance beyond the threshold, it is suggested that some of these MAGs could be part of the rare biosphere (Lloyd 2015; Lynch and Neufeld 2015). In addition to the low abundance, a lack of cultured representatives for most of these taxa catalogues them as part of the so‐called microbial dark matter (MDM). These lineages may also provide important functions in nutrient cycling. By the analysis of MAGs from microbial mats of Shark Bay it was proposed that MDM lineages use ribose, hydrogen and carbon dioxide as energy currencies to fill niches within the community (Wong et al. 2020). Similarly, MDM in microbial mats of Solar Lake, Sinai, showed genetic capacity for carbon fixation, degradation of complex polysaccharides and sulfur cycling (Abdallah et al. 2025). In a related scenario, several MAGs did not encode complete main metabolic pathways, yet they were part of the top members. In fact, these MAGs were associated with lineages described as symbiotic or parasitic (Brown et al. 2015), like Patescibacteria, Synergistota in the bacterial side or the archaea Micrarchaeota and Nanoarchaeota (Castelle et al. 2015). These microorganisms may provide intermediates or metabolites that facilitate otherwise unfavorable metabolisms; for example, the genus *Aminobacterium* of the phylum Synergistota was reported to ferment amino acids with production of acetate and hydrogen (Baena et al. 2000), which could be used by sulfate reducers or methanogens. Understudied alternative metabolic activities may also increase the competitiveness of specific taxa, like mobilization of heavy metals by Verrucomicrobia and Bacteroidetes in brackish microbial mats from Etang de Berre, South France (Vigneron et al. 2021) or by encoding biosynthetic genetic clusters as observed for KSB1 in microbial mats of Shark Bay (Chen et al. 2020). The new genomic knowledge described here may now inform other experimental strategies, such as incubation with specific substrates to achieve the enrichment of these taxa.

Although MAGs of the major and rare lineages were recovered, there is still more diversity in the microbial mats to be uncovered, as shown by the analysis of 16S rRNA gene sequences from the metagenomes. Other studies based on marker genes (García‐Maldonado et al. 2018; García‐Maldonado et al. 2023) and gene‐based shotgun sequencing (Maza‐Márquez et al. 2024) have reported a variety of lineages not recovered here as MAGs, showing that other and different strategies would be required for their genomic characterisation.

### 4.2 Encoded Metabolic Strategies

The analysis of the genetic content of the MAGs supported and expanded the potential metabolic capabilities of the community members. Because these genomes are in different degrees of completeness, the absence of specific genes should be taken with the same precaution as the inferred metabolism derived from the present genes. The metabolic potential identified in the MAGs, accompanied by their taxonomy, is in agreement with the main microbial guilds identified in Guerrero Negro (Des Marais 2003) and other microbial mats (Schneider et al. 2013; Wong et al. 2018). In the following section, we discuss the potential metabolism of the main players identified in this study.

Cyanobacteria of the family Coleofasciculaceae were the dominant potential primary producers, a finding that aligns with earlier research (Canfield and Des Marais 1991; Des Marais 2003). Although strains from Guerrero Negro are available in culture collections (García‐Pichel et al. 1998; Marter et al. 2025), to our knowledge, this is the first study where a genome has been generated. The availability of this resource may help to inform better subsequent analyses, like gene expression in specific conditions (Burow et al. 2013; Woebken et al. 2015). The other cyanobacterial genome, associated with Elainellaceae (Oculatellaceae, Mai and Johansen 2018), constitutes the first report of this taxon in Guerrero Negro, and was one of three cyanobacterial taxa found in microbial mats from Solar Lake (Abdallah et al. 2024). The main inferred metabolic pathways of cyanobacteria were in agreement with an oxygenic photosynthetic microorganism, which would reflect in both cyanobacterial MAGs negatively affected by the incubations.

The gamma‐Proteobacteria Beggiatoaceae showed genes related to dissimilatory sulfate reduction, which can operate in the reverse direction, oxidizing sulfur to sulfite (Schedel and Trijper 1979; Schedel et al. 1979; Crane 2019; Rudenko et al. 2024). While Beggiatoaceae was found enriched in the oxic zone, *Thiohalocapsa* was found to be highly abundant in all but the top layer. This contrasting vertical distribution pattern suggests niche separation between these potential thiosulfate oxidizers. The presence of anoxygenic photosystem II and *chlB* raises as the main features giving *Thiohalocapsa* a potential advantage in the anoxic zone of the mat (Imhoff et al. 2018).

The phylum Chloroflexota is known for its diversity and abundance in microbial mats (Nübel et al. 2001; Ley et al. 2006). Here, seven families were identified in five different vertical profiles and encoding three different potential carbon fixation strategies. Differences in potential for phototrophy and carbon fixation within the Chloroflexota are likely a result of different horizontal gene transfer events (Shih et al. 2017). Among Chloroflexaceae, the most common strategy for carbon fixation (3HP bi‐cycle) likely uses the acetate generated during the fermentative stages of cyanobacteria, with whom they seem to interact closely (Burow et al. 2013). The presence of genes for the CBB cycle detected in one Chloroflexaceae supports previous reports in Guerrero Negro of this puzzling feature (Pierson et al. 1994). Alternatively, the family B4‐G1, first found in Guaymas Basin (Dombrowski et al. 2018) and also reported in microbial mats of Shark Bay (Wong et al. 2018), encodes the WLP pathway for carbon fixation. Other potentially anaerobic fermentative families found within Chloroflexota were Anaerolineaceae, J111 with the capacity for carbon storage as polyhydroxyalkanoate (PHA), and SLSP01, which encodes a battery of cazymes. Of note, stimulated growth rates were reported for members of Anaerolineaceae when they are in co‐culture with hydrogenotrophic methanogens (Yamada et al. 2005, 2006). Genes for electron transfer enzymes, hydrogenases, motility, and transporters found in Chloroflexota could be involved in potential interactions with other taxa (Burow et al. 2013; Bunbury et al. 2025).

Bacteroidota showed significant diversity (8 bins) in microbial mats from Elkhorn Slough, California (Lee et al. 2018). Here, Bacteroidota was the third phylum with more representation (7 families). The Bacteroidota families were different from those commonly associated with near‐saturation ponds, the Salinibacteraceae, specifically the genus *Salinibacter* (Gómez‐Villegas et al. 2024) showing the high diversity and versatility of this phylum in hypersaline environments. Members of the phylum Bacteroidota are usually described as facultative aerobic heterotrophs and are associated with carbohydrate‐rich environments (Fernández‐Gómez et al. 2013). In microbial mats they are found in different layers (Ley et al. 2006; Farías et al. 2014; Wong et al. 2016), suggesting different roles and strategies for the degradation of organic matter. Bacteroidota from Guerrero Negro share with those of microbial mats from Shark Bay, the presence of the cyanophycinase gene, coding for an enzyme involved in the degradation of the cyanobacterial polymer (Wong et al. 2018). One Bacteroidota from Guerrero Negro was associated with the species *Salinivirga cyanobacteriivorans* (f_Salinivirgaceae). This species was found in a hypersaline microbial mat in the Kiritimati Atoll, and was described as a bacterium specialized in the degradation of cyanobacterial biomass in lower layers of the mat (Hania et al. 2017). For this role, *S. cyanobacteriivorans* encodes a variety of cazymes and peptidases. A complementary role was described in Bacteroidota isolates from a subsurface aquifer and a bioreactor, which were shown to scavenge dead cells through two different mechanisms; one isolate relied on cell‐to‐cell contact, while the second excreted lytic enzymes (Hirakata et al. 2023). Additionally, these isolates depended on symbiotic interactions with methanogens to maximize the degradation (Hirakata et al. 2023). Thus, Bacteroidota in Guerrero Negro mats may play similar roles in the degradation of complex matter generated by the major primary producers in the upper layers, while other members in the lower layers possibly scavenge dead cells with the possibility of increasing their degradation efficiency through interactions with methanogens.

The phylum Desulfobacterota was represented by eight families. Differences in genetic content for WLP, TCA, nitrogen fixation, and even their main feature the sulfate reduction, likely separate the niches occupied by MAGs of this phylum. Members of Desulfobacterota were found in different vertical profiles; this separation has been observed in Desulfobacterota of Kirimati Atoll microbial mats (Spring et al. 2019), likely reflecting potential metabolic differences. The family Desulfococcaceae contained genes for WLP but different completeness of the TCA cycle, indicating a versatility of carbon fixation metabolism within this family in order to completely oxidise acetate and other organic molecules to CO_2_. Two MAGs without WLP genes from families Desulfobacteraceae and Desulfoplanaceae, probably reduce sulfate using amino acids as electron donors and may convert the resulting acetate to CO_2_, a strategy suggested for Desulfobacteraceae in mangrove sediments (Qian et al. 2023). A less characterised taxon, the family BuS5, was described from marine carbon seeps from Guaymas Basin (Kniemeyer et al. 2007), where it showed complete hydrocarbon oxidation to CO_2_ and even preference for 12C‐alkanes. In Guerrero Negro, the BuS5 MAG presented potential for a versatile sulfur metabolism, suggesting that it is capable of reducing or oxidising thiosulfate. BuS5 could have the capability to use TMA, probably to release and use acetate; in this way, BuS5 may compete with methanogens for usage of this non‐competitive substrate. In support of this observation, the relatively high abundance of BuS5 in depths > 2 mm indicates that it is probably adapted to changes in redox conditions, while the methylotrophic methanogens are restricted to the anaerobic zone. A possible adaptation used for O_2_ detoxification would be *cydAB* genes, seen to be expressed under microoxic conditions (Lemos et al. 2001). These observations are in accordance with its high abundance (> 1%), seen preferably in the environmental sample from site A4N5.

The methanogenic archaea were associated with the methylotrophic lineage Methanosarcinaceae, recognised as the main methanogenic taxa in these hypersaline microbial mats (Orphan et al. 2008; García‐Maldonado et al. 2015; Kelley et al. 2015). Low abundance of methanogenic lineages may hinder their detection in microbial mats (Wong et al. 2018; Martínez‐Alvarez et al. 2023). Here, Methanosarcinaceae were highly abundant when the non‐competitive substrate TMA was used, reinforcing their known metabolic capabilities (Oren 2014). However, at the community level the methanogenesis was inferred as virtually absent in the environmental samples at both sites, as well as in the inferred metabolism of (environmental) mm‐scale samples, matching the low methanogenesis rates reported for Guerrero Negro (Bebout et al. 2004; Kelley et al. 2012).

Genes for bacterial aerobic methanotrophy, one of the metabolisms that would consume methane, were not detected in agreement with biogeochemical and molecular probe‐based reports (Bebout et al. 2004). Potential aerobic methanotrophic lineages previously reported by genetic markers include the bacterial phylum Verrucomicrobiota (García‐Maldonado et al. 2023). In our results, two MAGs of this phylum were recovered; nevertheless, they did not belong to the known methanotrophic lineage (Dunfield et al. 2007; Guerrero‐Cruz et al. 2021), and accordingly they do not encode the corresponding enzyme.

In turn, the anaerobic oxidation of methane is a metabolism much more complex to be ruled out since it can occur through reverse methanogenesis coupled to sulfate, nitrate and metals reduction through the same metabolic route of the hydrogenotrophic pathway (Hallam et al. 2004; Haroon et al. 2013; Bhattarai et al. 2019). Recently, signs of members of the archaeal lineages Methanophagales (ANME‐1) and Methanoperedenaceae (formerly ANME‐2d) were reported in these microbial mats through the gene‐marker approach (García‐Maldonado et al. 2023; Maza‐Márquez et al. 2024; Ramírez‐Arenas et al. 2024).

### 4.3 Genome‐Resolved Interpretation of Hypersaline Microbial Mats

The metabolic information of MAGs recovered from incubations was matched to the natural environment by analysing their vertical distribution in a public depth‐wise metagenomic dataset (Maza‐Márquez et al. 2022). This approach validates the MAGs by showing their presence in samples from different sites (A4N5 versus A5) or different temporalities (three‐year difference in sampling) in agreement with previous observations of temporal stability in the diversity of the microbial mats (Robertson et al. 2009). More importantly, the vertical distribution profiles were in agreement with the broad stratification, previously analysed by different methods (Ley et al. 2006; Dillon et al. 2009; Fike et al. 2008) and in general agreement with the stratification observed in microbial mats from the Kirimati Atoll (Schneider et al. 2013), Shark Bay (Wong et al. 2018), or from the Great Sippewissett marsh (Armitage et al. 2012). Moreover, the MAGs increase the taxonomic resolution known for the involved microorganisms. A clear example is Chloroflexota, which shows an even vertical distribution when analysed at the phylum level (Ley et al. 2006) and four different vertical distributions when MAGs are used for analysis.

Three out of six vertical distribution profiles identified expand across the four layers analysed. Thus, the differences in the abundance between layers separate the profiles. However, this community abundance profile may reflect the specific time the sample was taken (09:00 h) (Maza‐Márquez et al. 2022). It is well reported that diel vertical migration occurs in microbial mats and is associated with the shift in the chemical gradient (García‐Pichel et al. 1994; Spear et al. 2003). In this sense, Dillon et al. (2009) found migration in half of the peaks obtained by a terminal restriction fragment analysis in microbial mat samples taken at day and night times. The migratory taxa identified in that study included known motile *Beggiatoa* and *Microcoleus* (Coleofasciculus), several uncultured Bacteroidetes, Chloroflexibacteria (Chloroflexota), and deltaproteobacterium members. Following those reports, genes were found here for the motility types: gliding, (bacterial and archaeal) flagella, twitching, and phototaxis. The latter is present only in cyanobacteria. Besides adjusting their position to their environmental needs, these taxa may also participate in the translocation of nutrients, products and by‐products, otherwise inaccessible for non‐motile microorganisms. The presence of these phototrophic bacteria that generate organic matter, later available to lower layers, helps to sustain the diversity of anaerobic taxa (Ley et al. 2006; Harris et al. 2013; Lee et al. 2014). Together, the vertically migrating taxa promote diversity at the taxonomic level, and more importantly, at the metabolic level (Armitage et al. 2012). Alternatively, migration can also be used as a survival strategy in *Beggiatoa*, which usually resides at the oxygen–sulfide interface but in high sulfide flux conditions risks bursting from internal sulfur accumulation. In response, it may move downwards to the anoxic zone and switch its metabolism to reduce the internal sulfur to sulfide (Schwedt et al. 2012). It has also been suggested that a diel metabolic shift may affect the microbial fraction in the oxic zone while the microorganisms in the anoxic zone maintain their metabolic activity, although on more recalcitrant organic matter (Campbell et al. 2020). Supporting these functions, there were metabolic capabilities for the degradation of complex carbon between layers, and more specifically in taxa located in the anoxic layers harbouring a high count and variety of cazyme genes.

## 5 Conclusion

**FIGURE 7:**
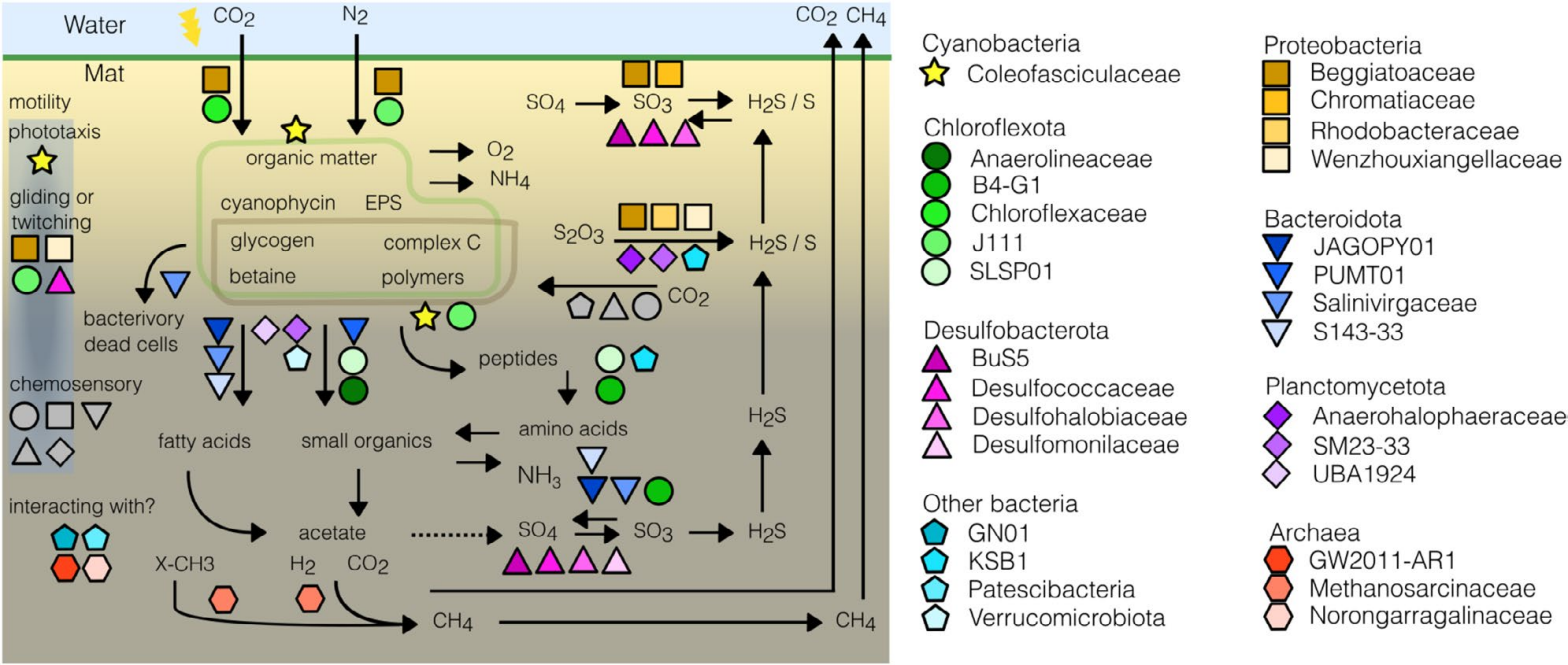
Conceptual overview of the main potential metabolic processes in hypersaline microbial mats. Shapes represent community members identified as main players in this study. Taxa are grouped by phylum (except for the ‘other bacteria’) and family names are shown. Shapes in grey are used to represent multiple members of the phylum.

Despite its previous intensive study, this work is the first genome‐resolved approach to the microbial community of hypersaline mats of Guerrero Negro. The sequencing of natural and incubated mat samples allowed us to recover MAGs that represent the main players of this widely diverse community. A conceptual overview of the potential metabolic roles by the main players in the Guerrero Negro hypersaline microbial mats is shown in Figure 7. Different metabolic capabilities were elucidated in the community members; supporting, confirming and linking previous observations by different biochemical and molecular methods, and revealing unexpected strategies in certain lineages. However, the metabolic potential of these genomes should be evaluated by other molecular techniques that allow the distinction of active members and pathways under specific conditions, like shotgun sequencing coupled with stable isotope probing (Malmstrom and Eloe‐Fadrosh 2019), or metatranscriptomics (Campbell et al. 2020). The recovered MAGs open new possibilities for the experimentation of interactions between community members and the exploration of rare and uncultured lineages. Altogether, these results show that the microbial communities of Guerrero Negro continue to be a source of knowledge and of multiple questions on the functioning of microbial communities.

## Author Contributions

**Miguel A. Martínez‐Mercado:** data curation, formal analysis, methodology, software, visualisation, writing – original draft. **Hever Latisnere‐Barragán:** data curation, investigation, methodology, validation, writing – review and editing. **Patricia J. Ramírez‐Arenas:** investigation, validation, writing – review and editing. **Ricardo Vázquez‐Juárez:** methodology, resources, writing – review and editing. **José Q. García‐Maldonado:** conceptualization, funding acquisition, investigation, methodology, project administration, writing – review and editing. **Alejandro López‐Cortés:** conceptualization, funding acquisition, investigation, methodology, project administration, resources, supervision, writing – review and editing.

## Conflicts of Interest

The authors declare no conflicts of interest.

## Supporting information


**Figure S1:** Ordination of k‐mer similarity among Guerrero Negro hypersaline microbial mat samples. Whole metagenome shotgun sequencing data was profiled based on k‐mers (k = 31) and similarities were analysed through ordination (MDS), the plot shows the first two components. Samples from Areas A4N5 and A5 were analysed untreated (Env) or after incubation in microcosm settings involving or not (Control) the substrates: H_2_/CO_2_, H_2_/CO_2_‐TMA, TMA (trimethylamine).

**Figure S2:** Community composition at the phylum level. Relative abundance of 16S rRNA gene sequences recovered from the metagenomes of environmental and incubated samples. Colours in bars represent phyla with relative abundance > 1% in at least one sample, the rest was collapsed into ‘Others’.

**Figure S3:** Top MAGs genomic content related to electron transfer enzymes. Circles indicate the presence of genes (for monomeric enzymes) or the extent to which completion of enzymatic complexes were achieved. White circles indicate absence. mtr, tetrahydromethanopterin S‐methyltransferase; fpo, F420H2 dehydrogenase; mvh, F420‐non‐reducing hydrogenase; hdrA2B2C2, Heterodisulfide reductase 2; hdr Heterodisulfide reductase subunit D and E; etfAB, electron transfer flavoprotein alpha and beta subunits; fixCX, electron transfer flavoprotein C and X subunits; hoxEFU, bidirectional [NiFe] hydrogenase; hoxHY, NAD‐reducing hydrogenase; hnd, NADP‐reducing hydrogenase; fdhB, formate dehydrogenase (coenzyme F420); fdhX, formate dehydrogenase (NADP+); fdo, formate dehydrogenase; bcd, butyryl‐CoA dehydrogenase; ldh, lactate dehydrogenase; rnf, H+/Na ± translocating ferredoxin:NAD + oxidoreductase; nqr, Na ± transporting NADH:ubiquinone oxidoreductase; hyaAB, hydrogenase; mbh, membrane‐bound hydrogenase.

**Figure S4:** Composition of Bacteria and Archaea taxa of intact and incubated microbial mat samples. Top MAGs were quantified and collapsed to the family level and their abundances were summed and represented as a heatmap. f_ = unclassified family; Env = intact samples; Control = incubated without substrate addition; TMA = trimethylamine.

**Figure S5:** Number of carbohydrate active enzymes (cazyme) per class in topMAGs. Cazyme classes: GH, glycoside hydrolase; PL, polysaccharide lyase; CE, carbohydrate esterases.

**Figure S6:** Peptidase genes in topMAGs. The number of genes with annotation as peptidases is shown for topMAGs. Peptidase types follow MEROPS classification (https://www.ebi.ac.uk/merops/index.shtml).

**Table S1:** Sample summary.
**Table S2:** Community composition by 16S rRNA gene.
**Table S3:** MAGs summary.
**Table S4:** Genes annotated in topMAGs.
**Table S5:** Cazyme genes in topMAGs.
**Table S6:** Peptidase genes in topMAGs.

## Data Availability

Sequence data generated in this study and reconstructed dereplicated Metagenome Assembled Genomes (MAGs) are openly accessible in the Sequencing Read Archive (SRA, https://www.ncbi.nlm.nih.gov/sra) under BioProject number PRJNA1086882.
