# Supplementary figures and images for: Genome‐Resolved Approach of Guerrero Negro Hypersaline Microbial Mats Reveals the Metabolic Potential of Key Players in a Stratified Community
Source: Environ Microbiol. 2025 Nov 5;27(11):e70199. doi: 10.1111/1462-2920.70199 (PMC12590107; doi:10.1111/1462-2920.70199)

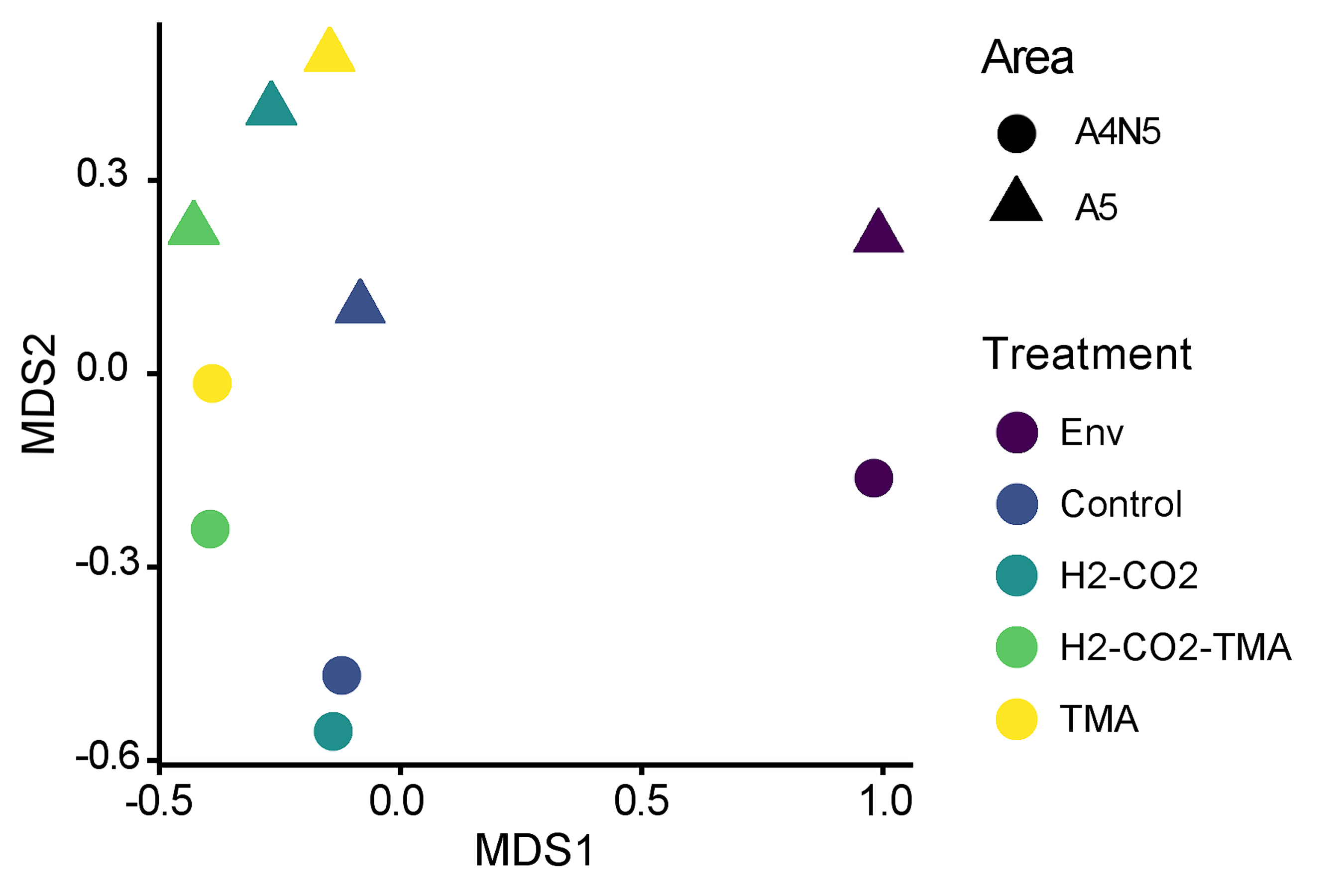

Supplement: Supplementary file 1 — Figure S1: Ordination of k‐mer similarity among Guerrero Negro hypersaline microbial mat samples. Whole metagenome shotgun sequencing data was profiled based on k‐mers (k = 31) and similarities were analysed through ordination (MDS), the plot shows the first two components. Samples from Areas A4N5 and A5 were analysed untreated (Env) or after incubation in microcosm settings involving or not (Control) the substrates: H2/CO2, H2/CO2‐TMA, TMA (trimethylamine). [file EMI-27-e70199-s006.tif]

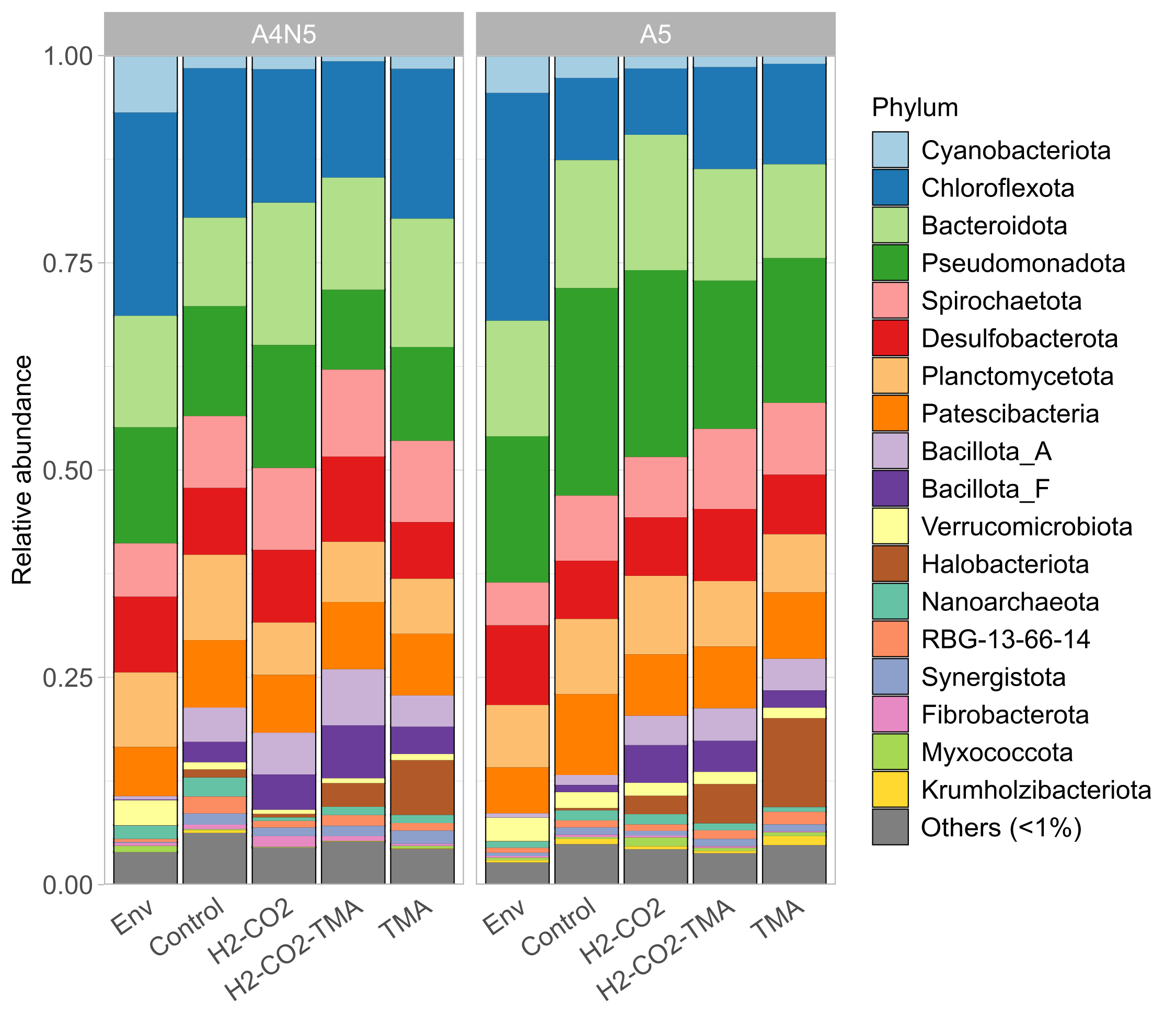

Supplement: Supplementary file 2 — Figure S2: Community composition at the phylum level. Relative abundance of 16S rRNA gene sequences recovered from the metagenomes of environmental and incubated samples. Colours in bars represent phyla with relative abundance > 1% in at least one sample, the rest was collapsed into ‘Others’. [file EMI-27-e70199-s005.tiff]

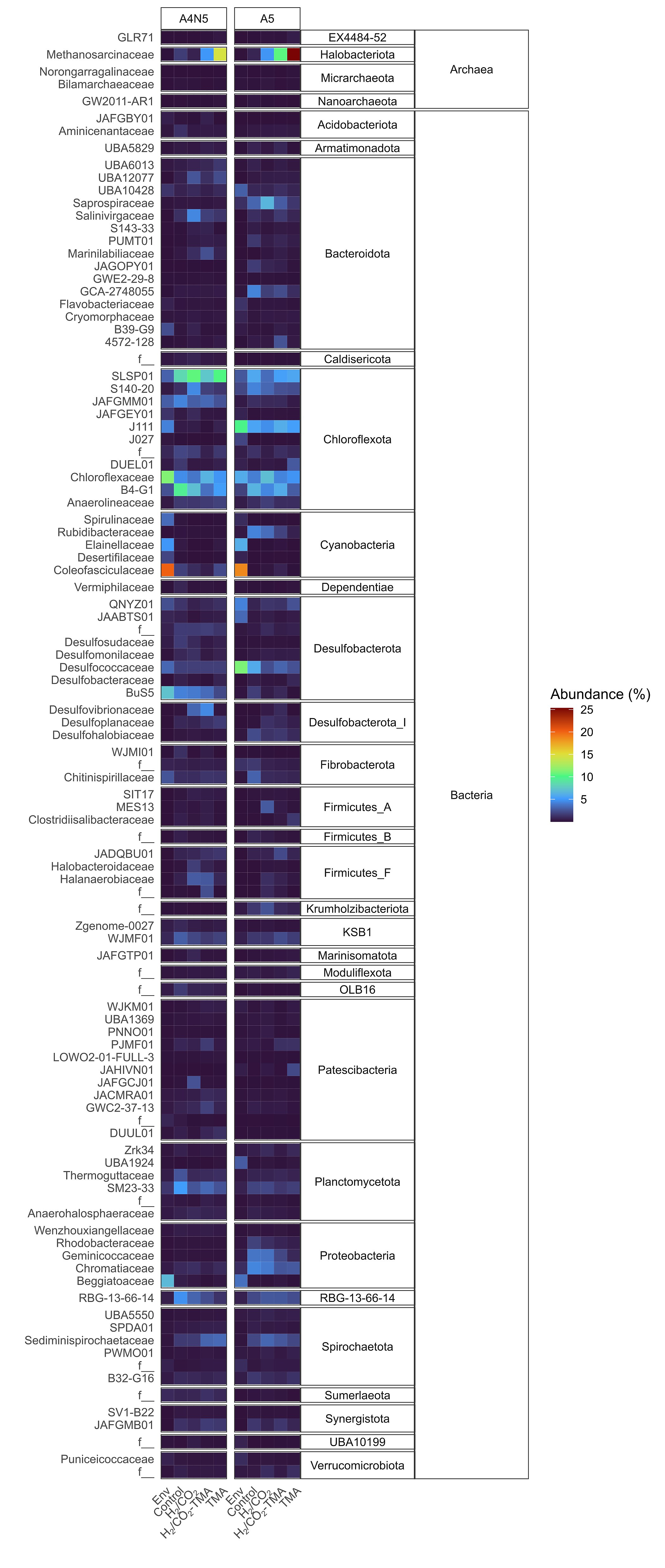

Supplement: Supplementary file 3 — Figure S3: Top MAGs genomic content related to electron transfer enzymes. Circles indicate the presence of genes (for monomeric enzymes) or the extent to which completion of enzymatic complexes were achieved. White circles indicate absence. mtr, tetrahydromethanopterin S‐methyltransferase; fpo, F420H2 dehydrogenase; mvh, F420‐non‐reducing hydrogenase; hdrA2B2C2, Heterodisulfide reductase 2; hdr Heterodisulfide reductase subunit D and E; etfAB, electron transfer flavoprotein alpha and beta subunits; fixCX, electron transfer flavoprotein C and X subunits; hoxEFU, bidirectional [NiFe] hydrogenase; hoxHY, NAD‐reducing hydrogenase; hnd, NADP‐reducing hydrogenase; fdhB, formate dehydrogenase (coenzyme F420); fdhX, formate dehydrogenase (NADP+); fdo, formate dehydrogenase; bcd, butyryl‐CoA dehydrogenase; ldh, lactate dehydrogenase; rnf, H+/Na ± translocating ferredoxin:NAD + oxidoreductase; nqr, Na ± transporting NADH:ubiquinone oxidoreductase; hyaAB, hydrogenase; mbh, membrane‐bound hydrogenase. [file EMI-27-e70199-s002.tif]

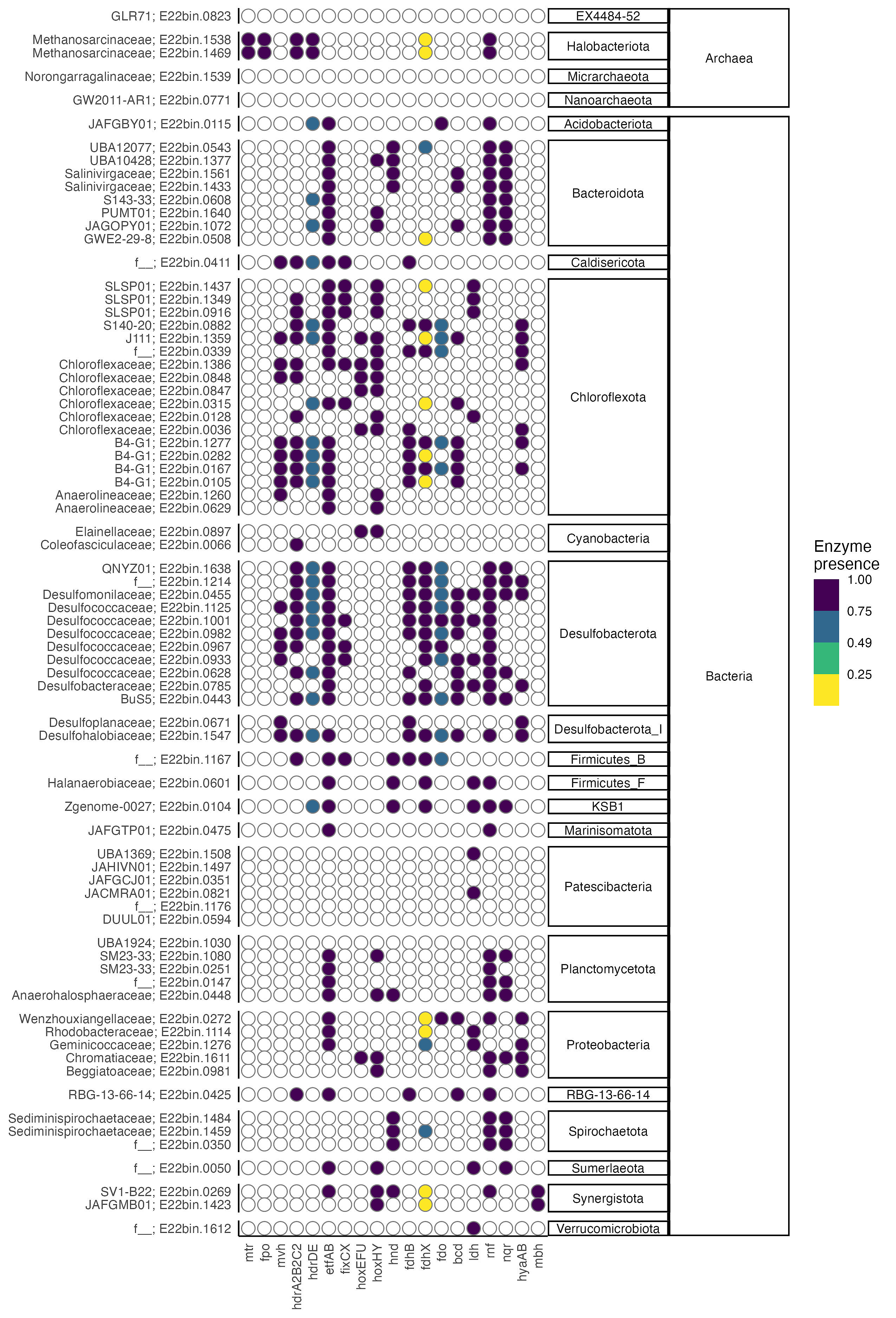

Supplement: Supplementary file 4 — Figure S4: Composition of Bacteria and Archaea taxa of intact and incubated microbial mat samples. Top MAGs were quantified and collapsed to the family level and their abundances were summed and represented as a heatmap. f_ = unclassified family; Env = intact samples; Control = incubated without substrate addition; TMA = trimethylamine. [file EMI-27-e70199-s004.tif]

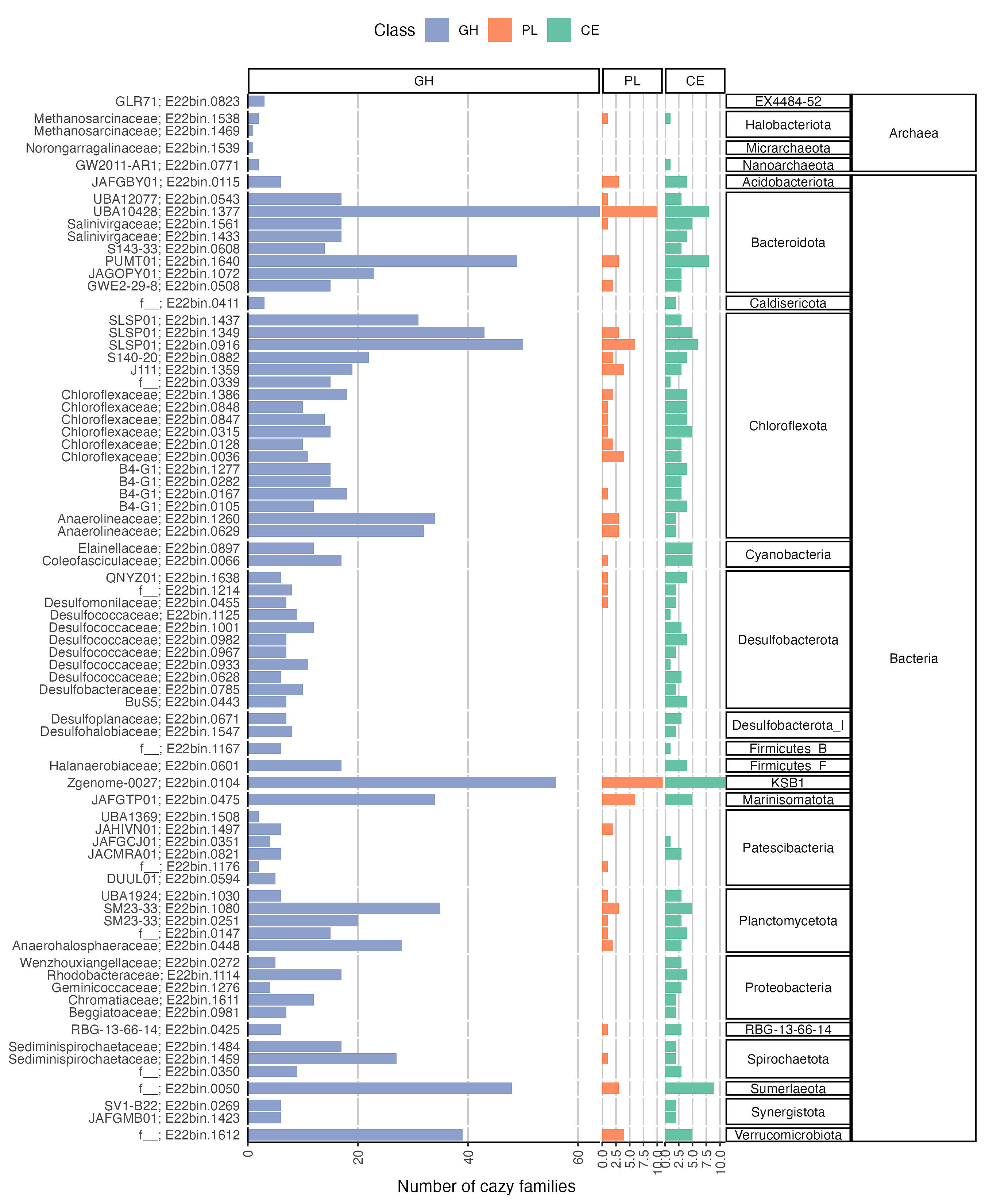

Supplement: Supplementary file 5 — Figure S5: Number of carbohydrate active enzymes (cazyme) per class in topMAGs. Cazyme classes: GH, glycoside hydrolase; PL, polysaccharide lyase; CE, carbohydrate esterases. [file EMI-27-e70199-s001.tif]

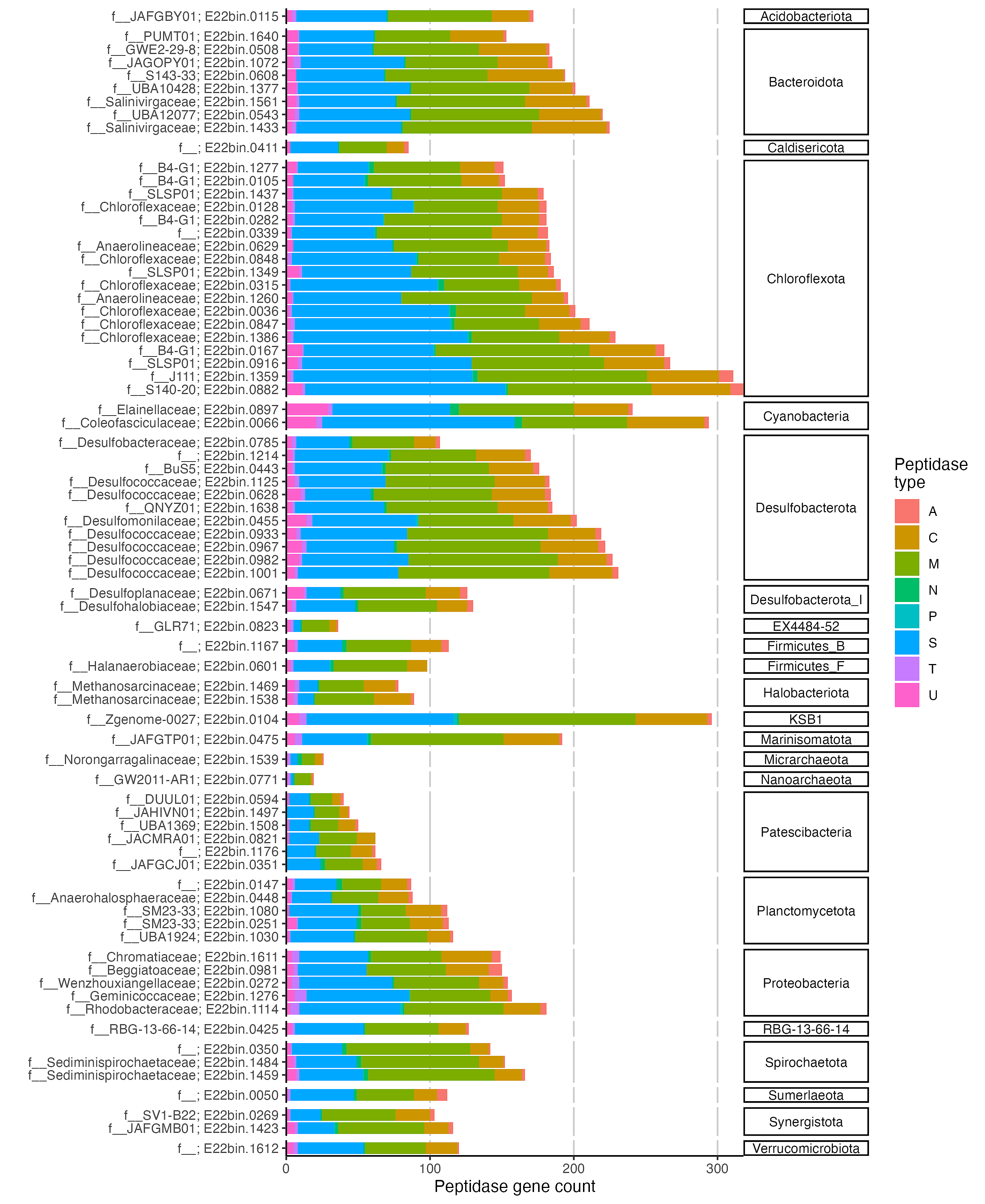

Supplement: Supplementary file 6 — Figure S6: Peptidase genes in topMAGs. The number of genes with annotation as peptidases is shown for topMAGs. Peptidase types follow MEROPS classification (https://www.ebi.ac.uk/merops/index.shtml). [file EMI-27-e70199-s007.tif]
